# Toward Real Chemical Accuracy on Current Quantum Hardware Through the Transcorrelated Method

**DOI:** 10.1021/acs.jctc.4c00070

**Published:** 2024-05-09

**Authors:** Werner Dobrautz, Igor O. Sokolov, Ke Liao, Pablo López Ríos, Martin Rahm, Ali Alavi, Ivano Tavernelli

**Affiliations:** † Department of Chemistry and Chemical Engineering, Chalmers University of Technology, 41296 Gothenburg, Sweden; ‡ IBM Quantum, IBM Research Zurich, Säumerstrasse 4, 8803 Rüschlikon, Switzerland; § Max Planck Institute for Solid State Research, Heisenbergstr. 1, 70569 Stuttgart, Germany; ∥ Yusuf Hamied Department of Chemistry, University of Cambridge, Lensfield Road, Cambridge CB2 1EW, U.K.

## Abstract

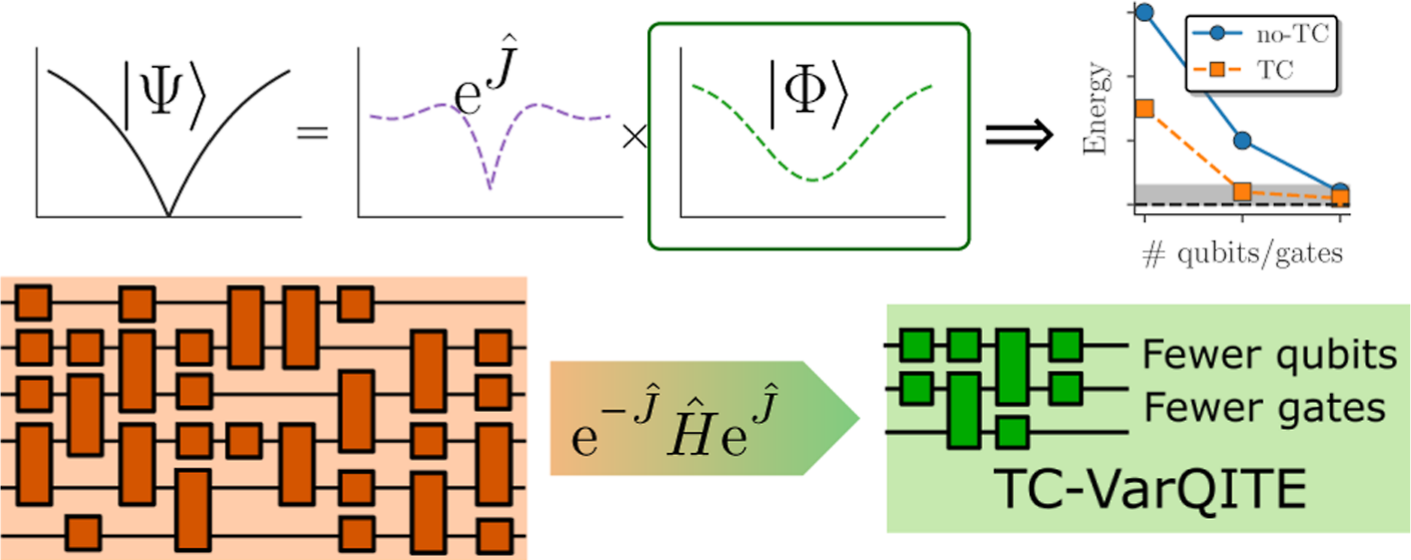

Quantum computing is emerging as a new computational paradigm with the potential to transform several research fields including quantum chemistry. However, current hardware limitations (including limited coherence times, gate infidelities, and connectivity) hamper the implementation of most quantum algorithms and call for more noise-resilient solutions. We propose an explicitly correlated Ansatz based on the transcorrelated (TC) approach to target these major roadblocks directly. This method transfers, without any approximation, correlations from the wave function directly into the Hamiltonian, thus reducing the resources needed to achieve accurate results with noisy quantum devices. We show that the TC approach allows for shallower circuits and improves the convergence toward the complete basis set limit, providing energies within chemical accuracy to experiment with smaller basis sets and, thus, fewer qubits. We demonstrate our method by computing bond lengths, dissociation energies, and vibrational frequencies close to experimental results for the hydrogen dimer and lithium hydride using two and four qubits, respectively. To demonstrate our approach’s current and near-term potential, we perform hardware experiments, where our results confirm that the TC method paves the way toward accurate quantum chemistry calculations already on today’s quantum hardware.

## 1 Introduction

Quantum computing^1,2^ has the potential for providing a significant speedup in the simulation of natural sciences compared to classical computational approaches. However, the implementation and application of quantum algorithms to relevant problems, e.g., in electronic structure theory, are still in its infancy. In this work, we show that the solution of molecular electronic structure problems using an explicitly correlated approach based on the transcorrelated (TC) method^3−7^ can take advantage of a quantum computing implementation. In fact, by enabling accurate and affordable quantum chemistry calculations for relevant problems, we argue that TC can become the method of choice for near-term demonstration of quantum advantage with state-of-the-art, noisy quantum computers.

Computational quantum chemistry is concerned with the solution of the electronic Schrödinger equation (SE) to obtain ground and excited state wave functions, their energies, and corresponding molecular properties.^8^ Sufficiently accurate modeling of the correlated motion of electrons would allow for the description of many groundbreaking yet unsolved physical and chemical phenomena, such as unconventional high-*T*_c_ superconductivity,^9^ photosynthesis,^10^ and nitrogen fixation.^11^ More generally, an efficient solver for the SE will make it possible to predict and design materials with novel and improved chemical and physical properties.

**Figure 1:**
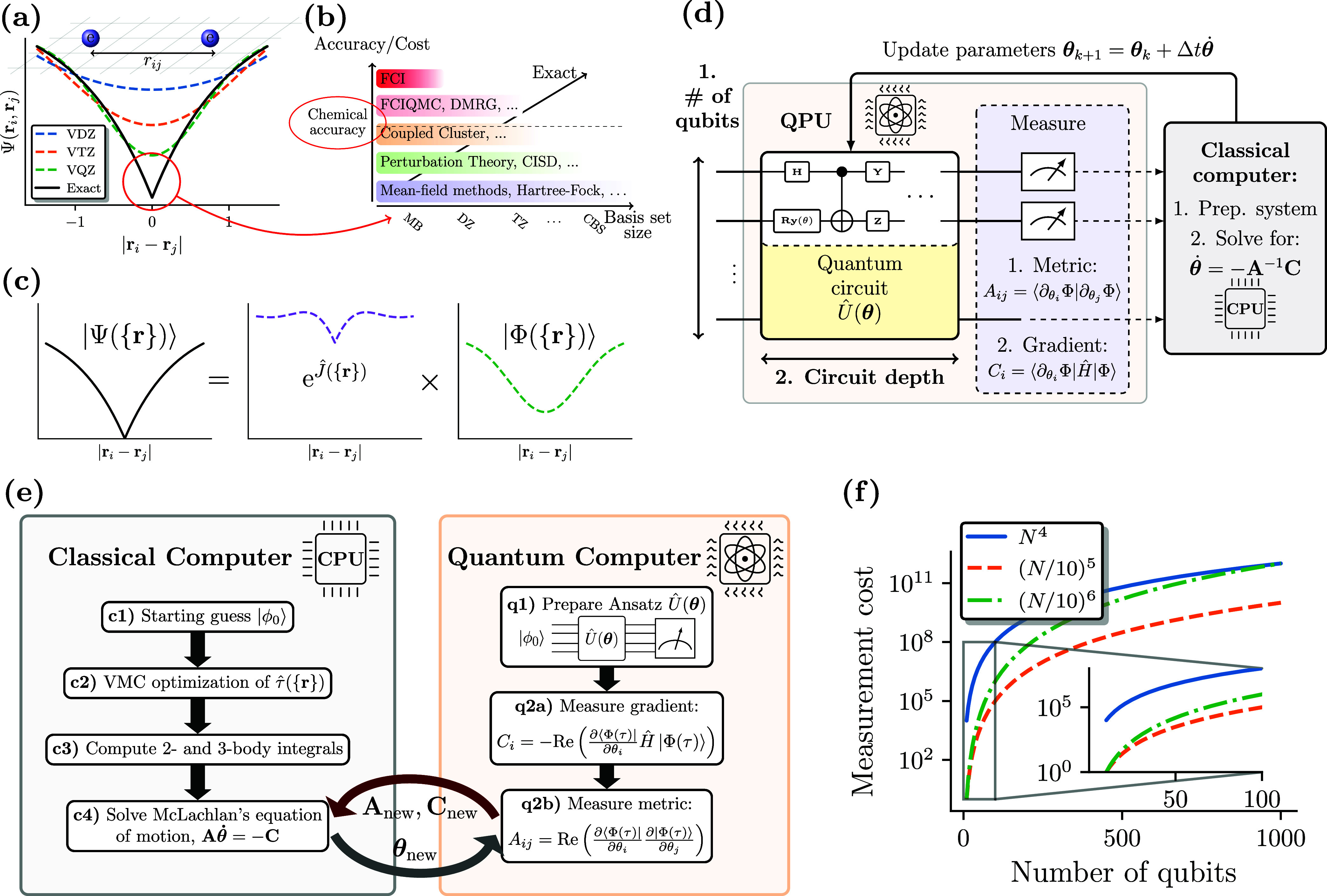
(a) Electronic cusp and increasing basis size to capture sharp features of the exact wave function. (b) Hierarchy of methods and basis size toward the exact complete basis set (CBS) solution. (c) Jastrow Ansatz to capture the cusp feature, leaving a smoother |Φ({**r**})⟩ to solve for. (d) VarQITE algorithm, where the metric, **A**, and the gradient, **C**, are measured on the QPU. The linear system of equations **A**θ̇ = −**C** is solved on a CPU to obtain the new parameters, which are fed back to the QPU. (e) Workflow of the TC-VarQITE approach to solve for the right eigenvector and groundstate energy of the TC Hamiltonian. On a CPU, we did the following: (c1) Perform a Hartree–Fock in a chosen basis set and optionally a MP2 calculation (using PySCF^75,76^ or OpenMolcas^77^ in this work) to obtain starting orbitals and |ϕ_0_⟩. (c2) Optimize the Jastrow factor with variational Monte Carlo (VMC). (c3) Compute the 2- and 3-body integrals for the subsequent electronic structure calculation. Then, we enter the quantum-classical optimization loop, sketched in (d), consisting of (q1) preparing a parametrized Ansatz and measuring the gradient (q2a) and the metric (q2b). (f) Measurements of operators containing 2-body and 3-body terms, scale as *N*^4^ (solid blue line) and *N*^6^, respectively, where *N* is the number of orbitals in the basis set. The TC method reduces the number of necessary orbitals by about an order of magnitude (green dash-dotted line). Neglecting 3-body excitations with six unique indices^59,60^ or mean-field approximations^78^ reduces the scaling of the TC method to the fifth or even the fourth power of *N* (with negligible errors in the systems studied in this work).

A wide variety of approximate quantum chemistry computational approaches have been developed, ranging from inexpensive mean-field Hartree–Fock (HF)^8^ to more reliable but expensive density matrix renormalization group (DMRG),^12^ coupled cluster (CC),^13^ and quantum Monte Carlo (QMC) methods.^14^ At their limit, i.e., in the absence of truncation and approximations, several such methods can approach the exact result, named the full configuration interaction (FCI) solution. FCI scales combinatorially with the number of electrons in a system and the size of the utilized *basis set expansion*; see Figure 1a,b.

The accuracy of typical quantum chemistry calculations is strongly affected by the quality of the *basis set*, which is used to expand the many-electron wave function in terms of one-electron basis functions.^8^ Such functions are commonly smooth Gaussian-type orbitals (GTOs),^15,16^ which produce tractable one- and two-body integrals, but fail to capture the electron-cusp condition.^17^ The cusp condition is a sharp feature of the exact ground state wave function induced by the divergence of the Coulomb potential at electron coalescence, which can typically only be captured through large basis sets. Using a larger number of basis functions results in a sizable increase in the required computational resources (see Figure 1a,b). Thus, more accurate methods are practically limited to small problem sizes even when using high-performance computing resources.

Quantum processors, on the other hand, harness quantum mechanical phenomena to potentially enable a significant leap in computation.^18^ By using *quantum bits* (qubits) as the basic unit of information and computation, quantum computers can encode exponentially growing problem sizes, 2^*n*^, into the Hilbert space of *n* qubits. Specifically designed quantum algorithms can then leverage wave function superposition and entanglement to solve certain classically challenging problems.^19^ Despite this potential, the sizes of quantum chemistry systems treatable on current noisy quantum hardware are still relatively modest and do not yet exceed the capability of conventional computing approaches. The main challenges to solve are qubit decoherence, gate noise, and the limited number of available qubits as, in the case of quantum chemistry, the number of qubits scales with the size of the required basis set. Thus, many methods to reduce the number of necessary qubits have been recently proposed. Among others, there are approaches leveraging system symmetries, others based on concepts such as entanglement forging,^20^ tensor hypercontraction,^21^ low-rank representations,^22^ methods for reducing the basis set size^23^ using Daubechies wavelets,^24^ basis-set-free solutions,^25^ or Hamiltonian downfolding techniques.^26−30^

Explicitly correlated methods,^31−36^ like the R12^37^ or F12 approaches,^38,39^ can reduce the need for large basis set expansions by directly incorporating the electronic cusp condition in the wave function Ansatz. Recently, it has been shown that methods based on these explicitly correlated approaches can yield accurate results already with relatively small basis sets and thus reduce the number of necessary qubits on quantum hardware.^23,40−43^ Motta et al.^23^ have used canonical transcorrelated F12 (CT-F12) theory^44−46^ to study several small molecular species, requiring far less quantum resource than conventional approaches. Kumar et al.^43^ extended CT-F12 to obtain accurate excited state energies with reduced quantum resources, and Schleich et al. used [2]_R12_ theory^47^ to a posteriori correct energy estimates obtained on quantum hardware.

In the TC approach,^4,6,7,48−58^ a correlated Ansatz–exactly incorporating the cusp condition–is applied and used to perform a similarity transformation of the electronic Hamiltonian, *Ĥ*, describing the ab initio chemical system. The undisputed benefit of the TC method is that it yields highly accurate results with very small basis set expansions^49,59,60^ and thus reduces the number of required qubits as well as the circuit depth on a quantum computer. The reduced circuit depth arises because the TC Hamiltonian has a more compact ground state,^41,42,48^ which can be accurately represented with shallower circuits.

The main challenge concerning implementing the TC approach is that the corresponding Hamiltonian is non-Hermitian. Most near-term quantum computing approaches rely on the minimization of the expectation value of a Hermitian operator (i.e., the energy as the expectation value of the Hamiltonian *Ĥ*) using the variational quantum eigensolver (VQE).^61^ To overcome this limitation, in this work, we use a variational Ansatz-based formulation of the projective quantum imaginary-time evolution (QITE),^62−66^ namely, the variational QITE (VarQITE) algorithm.^67,68^ With minor modifications, VarQITE enables the study of non-Hermitian problems,^41,42^ such as optimizing the TC Ansatz in a quantum computing setting.

The main differences between CT-F12 and the TC approach are (a) CT-F12 uses a unitary operator in the similarity transformation, which does not terminate naturally. Consequently, however, the transformed Hamiltonian remains Hermitian. (b) Two major approximations are used in CT-F12 theory. The Baker–Campbell–Hausdorff (BCH) expansion of the similarity transformation is truncated at the second order, and in the double commutator term, an effective 1-body Fock operator is used instead of the full Hamiltonian. The benefits of CT-F12 are that the Hamiltonian remains Hermitian and contains only up to 2-body terms. Additionally, by using a F12 Slater-type geminal,^38^ the spin dependence of the electron–electron cusp^69^ can be taken into account.^70^ Drawbacks include (i) that the BCH expansion in CT-F12 is truncated at the second commutator, and any higher-than-two-body interactions are ignored. This truncation induces errors that are not easy to eliminate, especially in the case of strong static correlations;^45,71^ (ii) the need to use a projection to ensure the orthogonality between the small and the augmented basis set, which can make the use of more sophisticated correlators, as used in our study, very complicated. As a result, CT-F12 uses a simpler correlated Ansatz and does not correct 1-body incompleteness,^43^ which can lead to worse results compared to TC approaches. The 1-body incompleteness was recently addressed in the work by Kumar et al.^43^ Opposed to the TC and CT-F12 approach, [2]_R12_ is an *a posteriori* correction, where one- and two-particle reduced density matrices, measured on quantum hardware, are used to improve final energy estimates.

To date, the largest quantum chemistry calculations performed on real quantum computers include Hartree–Fock calculations of a 12-atom hydrogen chain and a diazene isomerization^72^ along with correlated calculations of BeH_2_^73^ and H_2_O.^74^ The primary purpose of these calculations was to showcase the proof-of-concept of quantum computing using small basis sets rather than accuracy. In contrast, the TC method will pave the way toward accurate quantum chemistry calculations on quantum computers, allowing for precise calculations close to the CBS limit using small basis sets. Although explicitly correlated approaches, such as TC, significantly reduce the resource requirements for conventional methods such as FCIQMC or DMRG, practical problem sizes still quickly surpass those that these approaches can handle. We, therefore, believe that by reducing the number of qubits and gate operations the TC approach will make reliable quantum chemistry calculations of relevant problems with current and future error-mitigated quantum devices possible. Potentially surpassing the capabilities of conventional approaches in the future.

## 2 Theory and Algorithms

Within the Born–Oppenheimer approximation, the molecular Hamiltonian in first quantization (in atomic units, ℏ = |*e*| = *m*_*e*_ = 4πϵ_0_ = 1) is given by
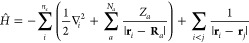
1

In eq 1, *n*_*e*_ is the number of electrons, *N*_*a*_ is the number of nuclei, **R**_*a*_ and *Z*_*a*_ are the position and atomic number of nucleus *a*, and **r**_*i*_ is the position of electron *i*. The divergence of the Coulomb potential, , induces a sharp cusp-like feature of the exact electronic wave function, |Ψ_0_(**r**)⟩, at electron coalescence, *r*_*ij*_ = |**r**_*i*_ – **r**_*j*_| = 0^17^ (Figure 1a). This sharp feature of |Ψ_0_(**r**)⟩ is challenging to capture using basis functions based on smooth GTOs and requires the use of large basis sets for accurate quantum chemical results (Figure 1b).

By introducing an explicit dependence on the electron–electron distances into the wave function via a Jastrow Ansatz,^79^, it is possible to exactly describe the nonsmooth behavior of |Ψ⟩, while leaving a much smoother wave function, |Φ⟩, to solve for (Figure 1c).  is an optimizable correlator depending on the positions of the electrons. The TC method^4^ incorporates this correlated Ansatz directly into the Hamiltonian of the system via a similarity transformation,
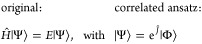
2
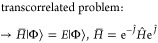
3

This similarity transformation removes the Coulomb singularity of the original molecular Hamiltonian, as shown in eq 1,^80^ and consequently increases the smoothness of the sought-after ground state wave function |Φ⟩.^81^ For the molecular Hamiltonian, eq 1, the TC Hamiltonian, , can be calculated exactly.^49^ possesses non-Hermitian two-body and three-body interaction terms.

In our applications, we use a Drummond–Towler–Needs Jastrow factor,^82,83^ which we optimize with VMC^84−86^ (with an efficient scaling of  on conventional hardware) using the CASINO package.^87,88^ We optimize the Jastrow factor by minimizing the variance of the TC reference energy, as proposed recently in ref (86). We then use the TC Hamiltonian integral (TCHint) library to calculate the 2- and 3-body integrals required to construct the molecular Hamiltonian in second quantization (Figure 1e). We want to point the interested reader to the recent ref (86), the Methods Section and the Supporting Information(89) for more details. Sample input files of the VMC optimization and integral calculation can be found in the Github repository accompanying the paper.^90^

Due to it being non-Hermitian, the TC Hamiltonian can theoretically yield energies below the exact CBS limit when using a finite basis set. However, since the TC Hamiltonian originates from a similarity transformation, the correct eigenvalues are obtained when using a large enough basis (approaching the CBS limit). The issue of nonvariationality has been thoroughly studied in ref (86) for the Jastrow factors and VMC optimization used in this work. It has been found that when optimizing the Jastrow factor by minimizing the variance of the TC reference energy, as done in this work, the results usually converge to the CBS limit from above. Additionally, in this and all recent studies using the TC approach (combined with a variety of methods and applied to different types of problems),^48,49,55−57,78,86^ the amount by which the TC results falls below the CBS limit is in all cases small enough to be safely ignored in practice.

Due to it being non-Hermitian, *H̅* has different left  and right , eigenvector solutions, which form a biorthonormal basis, . Another consequence of the non-Hermiticity of the TC Hamiltonian is that VQE cannot be used to solve for the ground state as the variational principle does not apply. To circumvent this obstacle, in this work, we solve for the right ground state wave function, |Φ_0_^*R*^⟩, (we drop the superscript “*R*” from here on), with the VarQITE algorithm.^41,68^ An additional benefit of the TC method is that the right ground-eigenvector of *H̅*, |Φ_0_⟩, has a more compact form compared to the non-TC ground state solution, |Ψ_0_⟩.^42,48^ Consequently, |Φ_0_⟩ is easier to prepare on quantum hardware with shallower circuits.^42^

QITE can be recast into a hybrid quantum-classical variational form (VarQITE)^41,68^ (Figure 1d), obtained by applying McLachlan’s variational principle to the imaginary-time SE
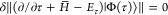
4where τ = *it* is imaginary time,  is the norm of a quantum state |Φ⟩, and  is the expected energy at time τ. With a parametrized circuit Ansatz, , with *n*_θ_ parameters, to represent/approximate the target right eigenvector, , of the TC Hamiltonian, eq 4 leads to a linear system of equations
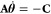
5which is solved on a classical computer. The updated parameters are obtained from θ(τ + Δ*τ*) = θ(τ) + Δτθ̇ for a chosen time-step, Δ*τ*. The vector **C** is composed of energy gradients, while the matrix **A** is related to the quantum Fischer information matrix or Fubini-Study metric^91^ and encodes the metric in parameter space of the Ansatz *Û*(θ). The VarQITE method can circumvent potential parameter optimization pitfalls,^91,92^ by a deterministic update of the circuit parameters according to eq 5. Both quantities **C** and **A** in eq 5 are sampled from the quantum circuit. This comes at the cost of  circuit evaluations to measure the matrix **A** at each iteration. However, accurate approximations^93^ have been proposed, which reduce the measurement scaling to linear,^91,94^ or even constant scaling,^95^ and it was recently shown by van Straaten and Koczor^96^ that the measurement cost of the gradient will dominate for large-scale quantum chemistry applications. The implementation of the VarQITE algorithm and the necessary modifications for non-Hermitian Hamiltonians (TC-VarQITE) are detailed in Methods Section.

The evaluation of the 3-body terms of the TC Hamiltonian might raise the question of scalability, as a 3-body term requires  measurements, where *N* is the number of basis functions/(spin-)orbitals in the basis set. However, one should consider that for an efficient implementation of the TC-VarQITE algorithm, one does not need an accurate evaluation of the energy (with all 3-body terms) at each iteration until convergence is reached. This can be monitored by calculating the norm ∥**A**^–1^**C**∥. Furthermore, as the TC method enables a faster convergence toward the CBS limit than conventional approaches, we expect an overall decrease in the number of orbitals *N* (and thus qubits) by an order of magnitude. This leads to a  scaling, i.e., to a decrease of the prefactor by 6 orders of magnitude. Overall, this reduction implies that in the regime up to 1000 qubits, the TC-VarQITE method entails orders of magnitude fewer measurements than in the non-TC case (see Figure 1f). Furthermore, recent studies^59,78,86^ show that the number of terms in the TC Hamiltonian can be reduced to (*N*/10)^5^ (by neglecting 3-body excitations with six unique indices^59^) or even to (*N*/10)^4^ by neglecting the pure normal ordered^97^ three-body operators and incorporating the remaining 3-body contributions in the two-, one-, and zero-body integrals^78^ (shifting the crossover far beyond 1000 qubits). The applicability of these types of approximations must be carefully considered for each studied system. However, the *N*^4^-scaling method introduced in ref (78) has recently been applied to the entire “HEAT” benchmark set^98^ and the *N*^5^ scaling approximation has been used in ref (59) for all first-row atoms, as well as the molecular systems CH_2_, FH and H_2_O, and in ref (55) for relative energies of molecular systems.

According to the work by Loaiza et al.,^99^ the 1-norm, ∑_*i*_|*c*_*i*_| of the coefficients, *c*_*i*_, of the linear combination of unitaries decompositions for molecular electronic structure Hamiltonians
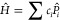
6is the main figure of merit associated with the quantum circuit complexity to measure the Hamiltonian. We measured the 1-norm of the coefficients corresponding to diagonal, one-, two- and three-body operators of the LiH (TC) Hamiltonian and compiled the results in Table S9 in the Supporting Information.^89^ We found that the normalized one-norm of the three-body operators, ∑_*i*,3-body_|*c*_*i*_|/∑_*i*_|*c*_*i*_|, is substantially smaller (below 0.1%) than the remaining contributions in the qubit Hamiltonians. Consequently, appropriate measurement cost reduction schemes^99−104^ can substantially lower the overhead due to the 3-body terms. Additionally, one can ameliorate the quantum computing measurement cost problem with approaches like informationally complete positive operator-valued measures^105−108^ classical shadows^109^ or shadow spectroscopy.^110^

Concerning the VMC optimization of the Jastrow factor, this considers only occupied orbitals in the initial HF solution. Virtual orbitals, constructed, e.g., from commonly used correlation-consistent basis sets^16^ are not optimized for the TC method. Following refs (111–113,) we will therefore use preoptimized natural orbitals (NOs) from second-order Møller–Plesset (MP2) perturbation theory calculations. In particular, orbital preoptimization works exceptionally well in conjunction with the TC method by efficiently truncating the virtual orbital space and reducing the resource (qubit) requirements further (see the Methods Section and the Supporting Information(89) for details). For a detailed comparison between the use of HF orbitals and MP2-NOs for LiH calculations, see the Sections IC and II of the Supporting Information.^89^ The overall workflow of the TC-VarQITE algorithm is shown in Figure 1e.

We want to summarize the additional computational cost of our proposed TC-VarQITE approach using VMC-optimized Jastrow factors and MP2-NOs compared with running VQE using HF orbitals. The baseline cost of VQE + HF is as a formally quartic scaling of HF with the number of orbitals, , (although practically the cost is usually closer to ) and a quantum measurement scaling of VQE of . These estimates assume “vanilla” implementations of HF (ignoring optimized implementations, i.e., using local approximation or density-fitting) and VQE (with no gradient information, optimized classical optimizers, or advanced grouping and measurement strategies). The additional cost of our proposed method is

- In the case of using MP2-NOs: Assuming a standard implementation, ignoring, i.e., local or density fitting/resolution of identity approximation, MP2 formally scales as .
- The optimization of the Jastrow factor using VMC has a square scaling, , in memory and a cubic time scaling with the number of electrons,  (ignoring, i.e., optimizations based on partitioning or subsampling).
- Ignoring any approximations to the 3-body terms, the calculation (time) and the storage (memory) of the TC integrals formally scale as .
- Measuring the metric for (TC−)VarQITE (ignoring any approximations) scales as  and measuring the gradient (with TC 3-body terms) scales as .

In quantum chemistry applications, the number of electrons is usually (much) smaller than the number of orbitals, *n*_*e*_ < *N*. Thus, the main increase in computational cost is the  scaling due to the TC 3-body terms. However, as argued above and shown in Figure 1f, the drastic reduction in the number of necessary orbitals (up to an order of magnitude in this work, *N* → *N*/10) due to the TC method outweighs this computational overhead in the range of up to 1000 qubits. Accurate approximations to the 3-body terms can reduce the scaling overhead of TC-VarQITE to , extending its range of advantage compared to “vanilla” VQE well beyond 1000s of qubits.^114−131^

## 3 Results and Discussion

We demonstrate the advantages of the TC approach with three applications on atomic and molecular systems: the beryllium atom and the hydrogen and lithium hydride molecules. If not specified differently, simulations are performed using HF orbitals and the unitary coupled cluster singles doubles (UCCSD) Ansatz,^132,133^ which gives reasonable indications about the performance of the TC method compared to the non-TC one. To demonstrate its potential, in this study, we initially solve the TC-VarQITE algorithm in the matrix formalism (statevector simulation), implying that all gates are implemented exactly (neither qubit decoherence nor gate infidelities are considered), and sampling noise is ignored. Up to 12 qubits, these noise-free results were obtained by simulation of quantum hardware. In contrast, data of larger calculations were obtained with a classical solver in the form of the TC-full configuration QMC (TC-FCIQMC) method.^48,49,134^

To enable hardware calculations, we also evaluated LiH with TC-VarQITE using a hardware-efficient Ansatz (HEA)^73^ and compared the results with non-TC calculations. Finally, to demonstrate the current and near-term hardware applicability of the TC approach, we calculated the LiH dissociation energy both with a noisy quantum circuit simulator and with actual hardware (HW) experiments on the 7-qubit ibm_lagos device.

Note that since the number of qubits needed to do a practical quantum computation is approximately equal to the number of spin–orbitals, in what follows, we use these two terms interchangeably. Details on the basis sets used in our calculations are provided in the figure captions and the Supporting Information.^89^

### 3.1 Beryllium Atom

**Figure 2:**
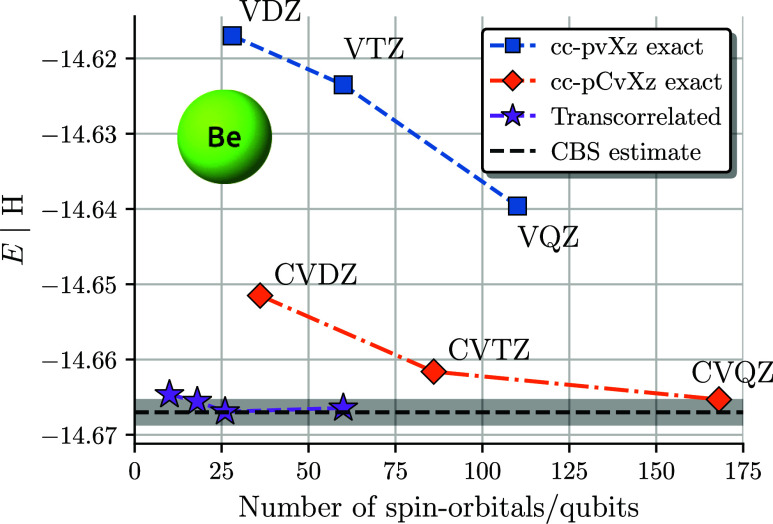
All-electron TC-VarQITE and non-TC FCI results for the beryllium atom using HF orbitals as a function of the number of spin–orbitals (or qubits). TC-VarQITE reaches chemical accuracy (gray area) of CBS limit estimates^135^ (black dashed line) using only 18 qubits.

Figure 2 shows all-electron TC-VarQITE results as a function of the basis set size for the beryllium atom. To achieve results within chemical accuracy compared to the CBS limit (i.e., 1 kcal/mol = 1.6 mHartree) (the gray area in Figure 2) with an FCI calculation, one would need 168 qubits, far beyond what can currently be used efficiently. The TC method, on the other hand, provides energies within chemical accuracy of the exact CBS limit while requiring only 18 qubits. This near-CBS accuracy shows the potential of utilizing an explicitly correlated method (without any approximation) in the form of the TC approach to enable near-term quantum devices to yield accurate results for relevant quantum chemical problems. We want to note that Schleich et al.^40^ have recently also obtained highly accurate results for the beryllium atom using small basis sets using the approximate VQE+[2]_*R*12_ explicitly correlated method.

### 3.2 Hydrogen Molecule

**Figure 3:**
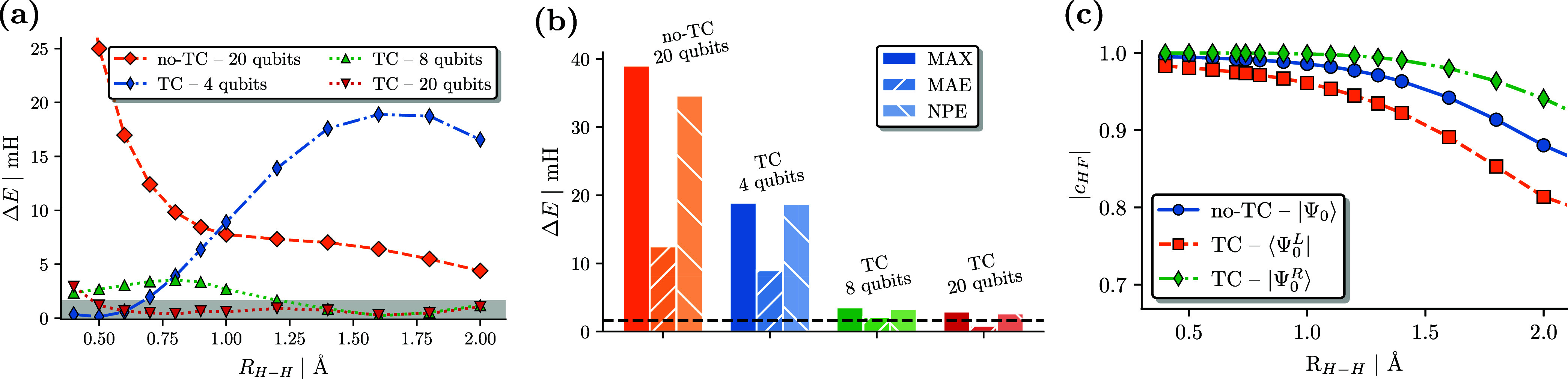
(a) Energy errors for TC calculations of the H_2_ bond dissociation w.r.t. CBS limit results (mH vs Å). TC-VarQITE calculations are based on HF orbitals in a STO-6G (4 qubits), 6-31G (8 qubits), and cc-pVDZ (20 qubits) basis sets. Also shown are no-TC FCI/cc-pVDZ calculations. The gray bar indicates chemical accuracy. (b) Error statistics in the form of the maximum error (MAX), the mean average error (MAE), and the nonparallelity error (NPE) for calculations shown in (a). (c) Hartree–Fock weight in the ground state wave function of the original Hamiltonian (no-TC), the left, ⟨Ψ_0_^*L*^| and right, |Ψ_0_^*R*^⟩, eigenvectors of the TC Hamiltonian, all computed in the cc-pVDZ basis set.

Figure 3a compares TC-VarQITE results for the H_2_ bond dissociation with CBS limit results. We also compare this to conventional FCI calculations in a cc-pVDZ basis set (corresponding to 20 qubits). TC-VarQITE results are shown for increasing basis set sizes using 4, 8, and 20 qubits, respectively. TC-VarQITE allows near chemically accurate results (with respect to the CBS limit, cf., gray area in Figure 3a) across the entire binding curve using only 8 qubits. It is noteworthy that whereas we reach near chemical accuracy with 20 qubits, conventional methods require at least 120 spin–orbitals for the same performance (see the Supporting Information(89)).

The additional benefit of increased compactness of the right eigenvector of the TC Hamiltonian^42,48^ can be appreciated in Figure 3c. The TC right eigenvector retains a more significant Hartree–Fock weight (*c*_HF_) and, thus, single-reference character across the whole H_2_ binding curve. Note how the increase of the *c*_HF_ component is particularly pronounced relative to the original ground state (no-TC) wave function in the strongly correlated dissociation regime, which is challenging for standard post-HF methods. Like the Hubbard model studied in ref (42) this increased compactness results in shallower circuit Ansätze for the ground state wave function.

### 3.3 Lithium Hydride

**Figure 4:**
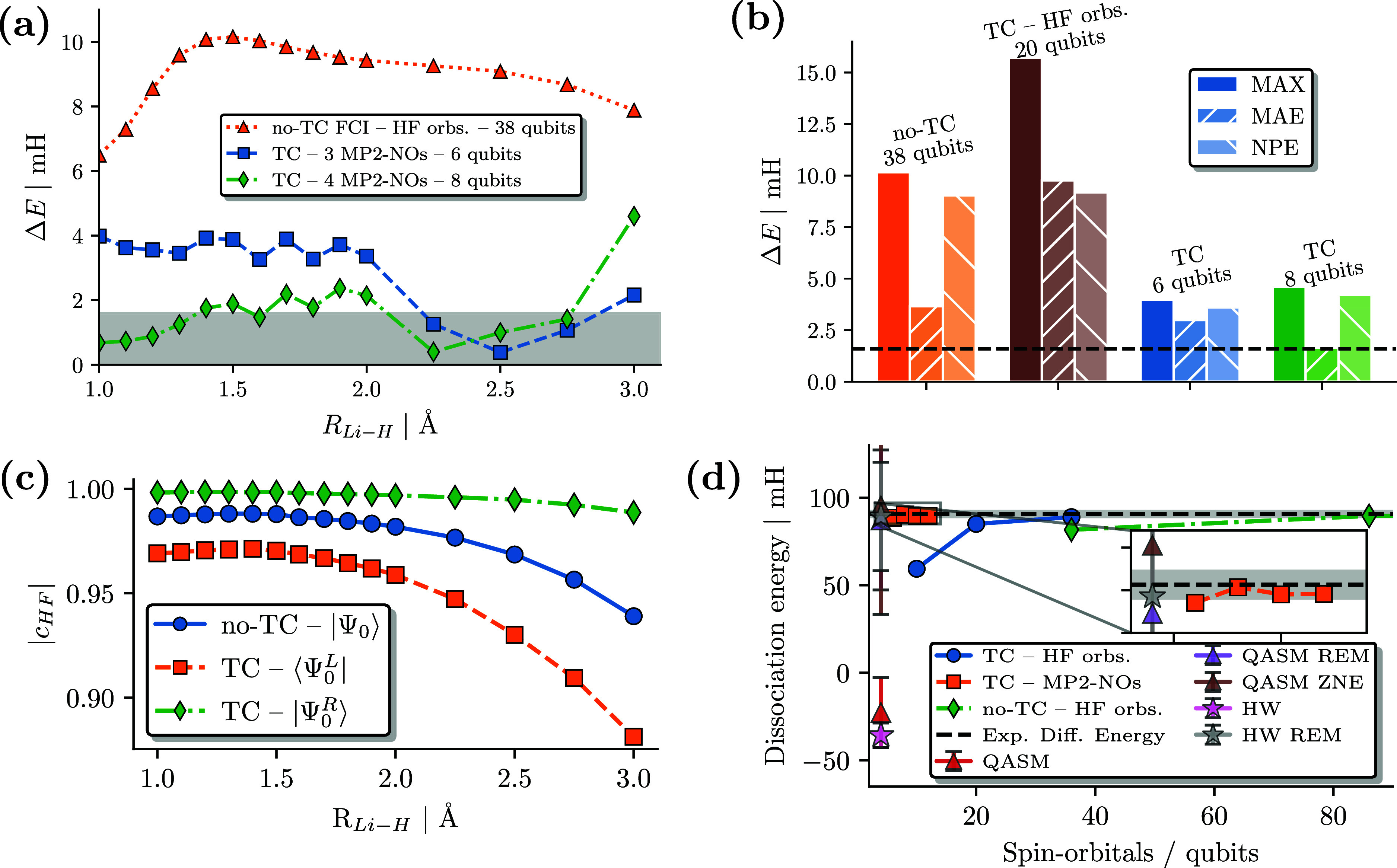
(a) Energy error of TC-VarQITE calculations using the three and four most occupied MP2-NOs in a cc-pVDZ basis for LiH w.r.t. CBS limit estimates in mH vs bond distance. We compare with no-TC FCI/cc-pVDZ (38 qubits) calculations using HF orbitals. (b) MAX, MAE, and NPE values for results shown in (a). (c) Hartree–Fock coefficient, *c*_HF_, of the all-electron ground state wave function using 14 MP2-NOs for the original Hamiltonian (no-TC) and the left, ⟨Ψ_0_^*L*^| and right, |Ψ_0_^*R*^⟩, eigenvectors of the TC Hamiltonian. Because the compactification of the right eigenvector is more pronounced for larger systems, a higher number of MP2-NOs are used to demonstrate this behavior. (d) LiH dissociation energy estimates (in mH) obtained with the TC method using HF orbitals in an STO-6G, 6-31G, and cc-pVDZ basis set (blue circles), MP2 NOs (orange squares), and conventional no-TC calculations (green diamonds) as a function of the number of spin–orbitals/qubits compared to experiment.^136,137^ QASM simulations and HW experiments on the ibm_lagos device are shown as triangles and stars, respectively. QASM and HW calculations used 3 MP2-NOs (4 qubits with parity encoding, see circuit in Figure 5b). Two independent error mitigation techniques [reference error mitigation (REM) and zero noise extrapolation (ZNE)] were applied to the noisy QASM/HW results. The gray bars indicates chemical accuracy.

Figure 4a-b shows the corresponding error analysis and comparison for the LiH molecule. TC-VarQITE provides drastically improved energies compared to conventional FCI/cc-pVDZ calculations (corresponding to 38 qubits). It is striking that it manages to do that by using only the four most occupied MP2-NOs (see the Methods Section and the Supporting Information(89)), requiring only 8 qubits on quantum hardware. TC-VarQITE yields results within or near chemical accuracy w.r.t. CBS limit (cf. gray area in Figure 4a) across the whole binding curve. The statistical error analysis shown in Figure 4b demonstrates how with just 3 or 4 MP2 NOs (corresponding to 6 and 8 qubits using a Jordan–Wigner Fermion-to-qubit encoding, respectively) TC-VarQITE readily outperforms conventional methods, even when these are leveraging more orbitals. Recently, Motta et al.^23^ and Kumar et al.^43^ obtained highly accurate results for H_2_ and LiH using small basis sets using the approximate CT-F12 explicitly correlated method.

**Figure 5:**
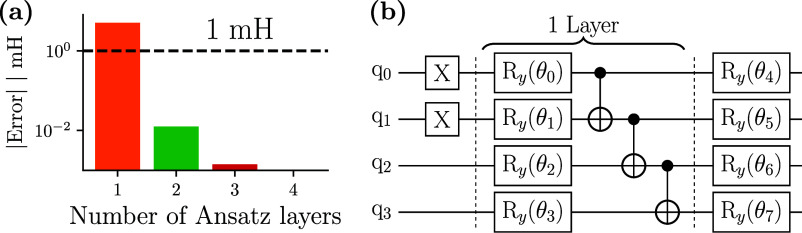
All-electron TC-VarQITE statevector simulations of LiH at equilibrium bond distance with 3 MP2-NOs. (a) Energy error (in mH) of the *R*_*y*_-Ansatz simulations w.r.t. the TC-FCI energy as a function of the number of used layers. Two layers of the *R*_*y*_-Ansatz suffice to obtain results within 1 mH of TC-FCI/cc-pVDZ using 3 MP2 NOs. (b) 1-layer *R*_*y*_-Ansatz with linear entanglement and a final rotation layer.

We note that in Figure 4c, the resulting “compactification” of the wave function (and corresponding circuit) is much more pronounced for LiH than for H_2_. This increased compactness suggests an increasing benefit of the TC approach for larger systems and exemplifies the favorable scalability of the method. With an HF coefficient greater than 0.99 over the entire dissociation profile, the TC right eigenvector can be efficiently mapped to exceedingly shallow quantum circuits suited for hardware calculations, as shown in Figure 5a.

With a TC Hamiltonian, we can calculate the dissociation energy of LiH within chemical accuracy to experiment with less than ten qubits (Figure 4d), a hardware cost that is compatible with experiments on current and near-term quantum devices. In contrast, no-TC methods would require a basis set as large as cc-pVTZ, corresponding to 88 spin–orbitals, to reach comparable results, as shown in Figure 4d.

To further substantiate the near-term potential of TC-VarQITE, we study the equilibrium bond distance of LiH using 3 MP2-NOs and a HEA.^73^ In this instance, we use repeated layers of *R*_*y*_ rotational gates (applied to each qubit) followed by linear entangling of CNOT gates (see Figure 5b). Parity encoding and 2-qubit reduction are also applied in this example.

Errors of this approach with respect to exact (state vector) UCCSD results are shown as a function of the number of Ansatz layers in Figure 5a. Already with two layers (16 single qubit R_*y*_ gates and 6 CNOT gates), the results are within 10^–3^ Ha from the UCCSD reference. To bring this result into perspective, not even a full cc-pVDZ basis (36 qubits with parity reduction) calculation would enable this level of accuracy with conventional methods (Figure 4c).

To test the hardware (HW) applicability of TC-VarQITE, we have applied it to calculate the LiH dissociation energy, which is known experimentally (Table 1). This calculation was done using a one-layer version of the HEA shown in Figure 5b, while initializing in the HF state, |Φ_HF_⟩, first in QASM simulations, then on HW (further details are provided in the Supporting Information). To account for the effect of noise, which causes raw QASM/HW results to be widely off the mark (Figure 4d), error mitigation was applied. We have separately tested two techniques: ZNE^138,139^ and REM,^140^ both alongside readout error mitigation, details of which can be found in the Supporting Information.^89^ Even though the standard deviations of our HW results are sizable due to noise, with error mitigation, TC-VarQITE yields QASM and HW predictions of the LiH dissociation energy close to (HW + ZNE) or within (HW + REM) chemical accuracy of the experimental results.

**Table 1:** Comparison With Experimental Data^136,141^^a^

	H_2_			
	qubits^b^	*R*_*e*_ (Å)	*D*_0_ (eV)	ω_*e*_ (cm^–1^)
no-TC^c^	2	0.7330	3.67	4954
	6	0.7462	3.87	4297
	18	0.7609	4.19	4353
CT-F12^f^	6	0.7397		4462
TC^d^	**2**	**0.7346**	**4.69**	**4435**
	**6**	**0.7428**	**4.66**	**4361**
exp.		**0.7414**	**4.52**	**4401**

	LiH			
	qubits	*R*_*e*_ (Å)	*D*_0_ (eV)	ω_*e*_ (cm^–1^)
no-TC^c^	10	1.5422	2.66	1690
	20	1.6717	1.80	1283
	36	1.6154	2.17	1360
CT-F12^g^	18	1.615		1385
TC^e^	**4**	**1.6032**	**2.42**	**1377**
	**6**	**1.5998**	**2.47**	**1390**
exp.		**1.5949**	**2.47**	**1405**

a Equilibrium distances (*R*_*e*_), dissociation energies (*D*_0_), and vibrational frequencies (ω_*e*_) are shown for H_2_ and LiH with and without TC. Results closest to *R*_*e*_ experimental data by Motta et al.^23^ using the CT-F12 method are also reported.

b With parity encoding and 2-qubit reduction.

c STO-6G, 6-31G, and cc-pVDZ basis sets.

d STO-6G and 631-G basis.

e 3 and 4 MP2-NOs. For more details, see the Supporting Information.^89^

f q-UCCSD/6-31G results of ref (23).

g q-UCCSD/comp results of ref (23).

### 3.4 Comparison with Experimental Data and Quantum Hardware Requirements

To further evaluate the TC-VarQITE approach, we calculated equilibrium bond lengths, *R*_*e*_, and vibrational stretching frequencies, ω_*e*_, (in addition to the above-studied dissociation energies, *D*_0_) for the H_2_ and LiH molecules and benchmark them against available experimental data (Table 1) as well as highly accurate CT-F12 results by Motta et al.^23^ We note excellent agreement for all spectroscopic quantities obtained with TC-VarQITE using only two qubits for H_2_ and four qubits for LiH and consistently equal good results using 6 qubits (see the Supporting Information(89) for details).

Estimates on the necessary quantum hardware requirements to obtain the results of Table 1 with TC-VarQITE are summarized in Table 2. We report the number of Ansatz parameters, the number of CNOTs, the total number of (1- and 2-qubit) gates, and the circuit depth–the number of quantum gates that cannot be executed simultaneously. We also show selected estimates of calculations without transcorrelation (no-TC) and available data by Motta et al.^23^ using the CT-F12 approach. All our estimates use parity Fermion-to-qubit encodings (with a subsequent 2-qubit reduction) and all but one use the default UCCSD implementation of Qiskit.^142^ The entry indicated by TC + HEA employs a 2-layer hardware efficient R_*y*_ Ansatz with linear entangling shown in Figure 5b that yields sub microhartree precision for LiH at equilibrium bond distance (Figure 5a).

**Table 2:** Estimate of Quantum Circuit Requirements for the Calculation of Spectroscopic Constants in Table 1 Using Parity Encoding With 2-Qubit Reduction and a Default UCCSD Ansatz (Except the Last Row)^a^

system	basis	orbitals	qubits	method	Paulis^b^	parameters	gates	CNOTs	depth
H_2_	STO-6G	2	2	no-TC	5	3	21	4	14
H_2_	STO-6G	2	2	**TC**	**7**	3	21	4	14
H_2_	6-31G	4	6	no-TC	159	15	1271	560	779
H_2_	6-31G	4	6	**TC**	**607**	15	1271	560	779
H_2_	6-31G	4	6	CT-F12^c^	235	15	741	476	604
LiH	cc-pVDZ	18	36	no-TC^c^	−	323	110,230	89,080	95,507
LiH	6-31G	10	18	CT-F12^c^	8527	99	12,087	9644	10,780
LiH	**MP2-NOs**	**3**	**4**	**TC**	**108**	**8**	**430**	**172**	**275**
LiH	**MP2-NOs**	**3**	**4**	**TC + HEA**^d^	**108**	**12**	**20**	**6**	**10**

a Number of parameters of the quantum circuit Ansatz, number of two-qubit CNOTs and the total number of gates (obtained with Qiskit’s count_ops() function), as well as circuit depth (sets of quantum gates that cannot be executed simultaneously). Data for CT-F12 calculations are taken from ref (23).

b Terms smaller than 10^–8^ Ha in absolute value are omitted.

c From ref (23).

d Using a 2-layer hardware efficient R_*y*_ Ansatz with linear entangling shown in Figure 5b that yields sub microhartree precision for LiH at equilibrium bond distance (Figure 5a).

The results of Table 2 demonstrate the drastic reduction in the necessary quantum resources, not only in the total number of qubits but also in the required circuit depth. TC-VarQITE results for H_2_ using an STO-6G basis (two qubits with parity encoding) need only 4 CNOT gates and a circuit depth of 14 to yield results closer to experiment than no-TC or CT-F12 calculations^23^ requiring over 400 CNOTs. The powerful combination of TC with MP2-NOs is demonstrated for LiH. TC-VarQITE using 3 MP2-NOs (4 qubits with parity encoding) requires only 172 CNOTs and a circuit depth of 275 to yield highly accurate spectroscopic data. Alternative approaches (no-TC or CT-F12) need larger basis sets, more qubits, and much deeper circuits to achieve a similar accuracy. Using hardware-efficient Anätze further drastically reduces the TC-VarQITE hardware requirements to only 6 CNOT gates and a quantum circuit depth of 10.

On the other hand, Table 2 also demonstrates the drawback of the TC approach in the form of the increased number of Pauli terms due to the 3-body in the Hamiltonian, as shown in eq 2. That is, the H_2_ TC Hamiltonian using a 6-31G basis (6 qubits with parity encoding) has 607 terms compared to the 235 terms of the CT-F12 and 159 terms of the original (no-TC) Hamiltonian. However, with TC-VarQITE, using an STO-6G basis, and thus only 7 Pauli terms, suffices to reach the same accuracy as other methods in larger basis sets.

## 4 Conclusions and Outlook

This paper describes a quantum computing implementation of an explicitly correlated method based on the TC approach. The TC method drastically reduces the number of required qubits and circuit depth to obtain results within chemical accuracy to experiment. Here, we consider the exact TC formalism and propose efficient theoretical and computational solutions to overcome the challenges preventing its implementation on near-term quantum computers.

By incorporation of the electron cusp condition, the TC method approaches CBS limit results and enables chemically accurate calculations with relatively small basis set sizes. Because the TC Hamiltonian is non-Hermitian, it cannot be directly combined with the conventional VQE. To overcome this issue, we made use of the variational (Ansatz-based) QITE algorithm (VarQITE),^68^ for which recent advances^42^ enable an efficient extension to non-Hermitian problems. In addition, we employ a preoptimized set of NOs obtained from second-order Møller–Plesset perturbation theory calculations^111^ (MP2-NOs) to efficiently truncate the virtual orbital space. MP2-NOs work exceptionally well in conjunction with the TC method and help to further reduce the number of required qubits.

We demonstrate the TC-VarQITE approach, combined with orbital optimization, on small atomic and molecular test systems including the beryllium atom, the hydrogen dimer, and lithium hydride. In all these cases, we could closely reproduce experimental values, including bond lengths, dissociation energies, and the vibrational frequencies of H_2_ and LiH, using just two and four qubits, respectively. Finally, to illustrate the applicability of the TC-VarQITE approach in quantum hardware experiments, we also evaluated the bond dissociation energy of LiH. When combined with error-mitigation techniques, our hardware results show a great level of accuracy close to the CBS limit and spectroscopic data. The mitigation techniques include ZNE,^138,139^ reference-state error mitigation,^140^ together with the commonly used readout error mitigation.^143^

The aim of this work was the implementation and demonstration of the prowess of the unapproximated TC approach to ab initio molecular on quantum hardware. To do this, we chose what might be considered “minimal” test systems. However, as has been done on “conventional” hardware,^49,55,57,59,78,86^ in future work, we will extend the application of TC approach to larger molecular systems than studied here. Additionally, we will develop new methodologies to obtain not only energy estimates, but also properties in the form of unbiased density matrices, and consequently, combine the TC approach with self-consistent orbital optimization.^144−148^/embedding,^149−152^ spin-conserving schemes,^153−157^ as well as adaptive quantum circuit Ansätze.^158−161^

In conclusion, the full potential of the TC method manifests as a dramatic cost savings (in terms of the number of qubits and circuit depth) for current quantum hardware calculations. Our study demonstrates that TC-VarQITE has the potential to become the method of choice for calculating accurate quantum chemistry observables of relevant molecular systems on current and near-term quantum computers.

## 5 Methods

### 5.1 Transcorrelation

Transcorrelation is the application of a similarity transformation to the SE of a system, *Ĥ*Ψ = *E*Ψ, to absorb the Jastrow factor  from the Ansatz  into an effective Hamiltonian. The resulting TC SE, , can be solved in second quantization using any quantum chemistry eigensolver, including quantum computers, with the advantage that the FCI solution for Φ is much more compact than that for Ψ and thus easier to represent approximately. Eigensolvers only require the values of the matrix elements of  between different determinants. If the Jastrow factor can be written as *J* = ∑_*i*<*j*_*u*(**r**_*i*_, **r**_*j*_) then
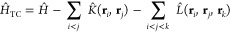
7where  is an operator that modifies the values of two-electron matrix elements and introduces non-Hermiticity and  is an operator that connects determinants separated by triple excitations. Eigensolvers thus need the ability to accommodate non-Hermiticity and three-electron matrix elements, so non-TC implementations usually require some degree of modification.

We use a Drummond–Towler–Needs Jastrow factor,^82,83^ which we optimize with VMC^84−86^ (with a scaling of  on conventional hardware) using the CASINO package.^87,88^ We optimize the Jastrow factor by minimizing the variance of the TC reference energy, as proposed recently in ref (86). We then use the TCHint library to calculate the 2- and 3-body integrals required to construct the TC molecular Hamiltonian in second quantization. See ref (86) and the Supporting Information for more details and sample input files of the VMC optimization and integral calculation can be found in the Github repository accompanying the paper.^90^

### 5.2 Variational Ansatz-Based QITE

The VarQITE algorithm^68^ is based on McLachlan’s variational principle, which is used to derive the evolution of gate parameters, represented by **θ**(τ), for a wave function Ansatz. The derivation is encapsulated in eq 4 of the main text, which leads to a linear system of equations defined in eq 5 of the main text. This system necessitates the computation of matrix elements associated with the matrix **A** and the gradient vector **C** defined as
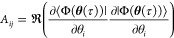
8and
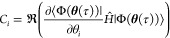
9where the wave function Ansatz is differentiated with respect to the gate parameters. In our implementation, their calculation is performed using the methodology outlined in ref (42), specifically designed for non-Hermitian (TC) problems. Next, we give more details about the steps necessary to reproduce the results of this work.

In numerical simulations, the values of *A*_*ij*_ and *C*_*i*_ are estimated using the forward finite-differences method^162^ given by
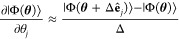
10where  is *j*-th element of the *n*_θ_-dimensional unit vector. We chose a step size of Δ = 10^–3^ in this work. To generate the state-vector representation of the Ansatz, |Φ(**θ**)⟩, we create the corresponding quantum circuit in Qiskit^142^ and then convert it to a state vector. This approach allows for the incorporation of gate errors through realistic noise models of IBM Quantum processors. The matrix elements *A*_*ij*_ and *C*_*i*_ can be computed independently, and we parallelize their computation on multiple CPUs by using the ipyparallel library to speed up our simulations. Although the forward finite-differences method provides satisfactory results for the computation of the derivatives, the parameter-shift rule^163^ can also be employed within our framework to obtain analytic derivatives.

In hardware calculations, the matrix elements *A*_*ij*_ and *C*_*i*_ are calculated via the differentiation of general gates by means of a linear combination of unitaries.^163^ To compute the *C*_*i*_ elements, we use the quantum circuit shown in Figure 1. For a Hermitian Hamiltonian, a Hadamard gate (*H*) is applied before measuring the ancilla. For a non-Hermitian Hamiltonian, *H̅*, we decompose *H̅* into Hermitian and anti-Hermitian components denoted by , where  and . Subsequently, the circuit from Figure 1 is applied to each term of  and , where an  rotational gate is applied instead of a Hadamard in the case of . This circuit’s measurement outcomes are combined to obtain *C*_*i*_ as in refs (42 and 163) To compute the *A*_*ij*_ matrix elements, we proceed in the standard way, which can be found in ref (19) since they are independent of the Hamiltonian. We typically use 10^4^ to 3.2 × 10^4^ shots (measurements) to collect enough statistics to accurately estimate the expected values.

For the representations of Ansatz circuits, we use Qiskit’s implementation of UCCSD and hardware-efficient Ansätze with the default settings.

Having all the necessary quantities, the linear system in eq 5 of the main text can be approximately inverted to obtain  using the least-squares solver (default settings) implemented in SciPy.^164^ Finally, the updated parameters are obtained from θ(τ + Δ*τ*) = θ(τ) + Δτθ̇ for a chosen time-step of Δ*τ* = 0.05 in this work.

**Figure 6:**
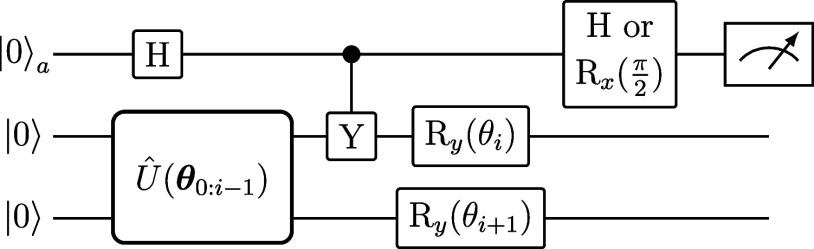
Quantum circuit used to calculate the *C*_*i*_ term in the (non-)Hermitian case for a (TC) Hamiltonian. A Hamiltonian is first separated into its Hermitian and anti-Hermitian parts. The circuit uses a Hadamard gate, *H*, for the Hermitian part and an  rotational gate for the anti-Hermitian part before measuring the ancilla. This circuit needs to be repeated for every term in a Hamiltonian.

Figure 6 shows the quantum circuit used to calculate the *C*_*i*_ term in the (non)Hermitian case for a (TC) Hamiltonian.

### 5.3 Second Order Møller–Plesset NOs (MP2-NOs)

The 1-body reduced density matrix (1-RDM) in a second-quantized basis is defined as
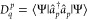
11where |Ψ⟩ is the wave function and  is the Fermionic annihilation(creation) operator of an electron in orbital *q*(*p*) of the current basis. The diagonalization of eq 11 provides eigenvalues in terms of the occupation numbers and eigenvectors that correspond to the transformation matrix from the current basis to the NO basis. Löwdin^165^ first used NOs to accelerate the convergence of configuration interaction calculations by retaining only those NOs with significantly nonzero occupation numbers. The specific NOs used in this work are obtained on the MP2 level. First, a mean-field Hartree–Fock (HF) solution to the system under study is obtained in a reasonably large basis set, e.g., cc-pVDZ or cc-pVTZ. In the HF canonical orbital basis, the MP2 wave function is
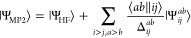
12where we follow the convention to use *a*, *b*, ... and *i*, *j*, ... to indicate the unoccupied (virtual) and occupied spin–orbitals, respectively. The antisymmetrized Coulomb integrals are defined as ⟨*ab*∥*ij*⟩ = ⟨*ab*|*ij*⟩ – ⟨*ab*|*ji*⟩ and the denominator is  = ε_*i*_ + ε_*j*_ – ε_*a*_ – ε_*b*_ with ε denoting orbital energies (diagonal elements of the Fock matrix).

Plugging |Ψ_MP2_⟩ into eq 11, we find
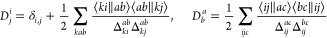
13where we ignore orbital rotations between the occupied and virtual space by setting the occupied-virtual block  =  = 0. In literature,^111−113^ the so-called frozen natural orbitals are obtained by only diagonalizing the virtual–virtual block of the 1-RDM . In this work, we diagonalize both the occupied–occupied and virtual–virtual blocks.

## Supporting Information Available

Data and software to reproduce this work are available in an accompanying public Github repository.^90^. Further details and results concerning the computational workflow and settings for the beryllium, H_2_ and LiH calculations; the use of MP2 NOs; the H_2_ CBS limit extrapolation; the calculation of the LiH dissociation energy and spectroscopic constants; the QASM simulations/quantum hardware calculations and error mitigation schemes; and the 1-norm of the TC qubit Hamiltonian (PDF)
