# Supplementary material for: Toward Real Chemical Accuracy on Current Quantum Hardware Through the Transcorrelated Method
Source: J Chem Theory Comput. 2024 May 9;20(10):4146–60. doi: 10.1021/acs.jctc.4c00070 (PMC11137825; doi:10.1021/acs.jctc.4c00070)
Supplement: Supplementary file 1 — ct4c00070_si_001.pdf [file ct4c00070_si_001.pdf]

Supporting Information : Towards real chemical accuracy on current quantum hardware through the transcorrelated method

Werner Dobrautz* and Martin Rahm

Department of Chemistry and Chemical Engineering, Chalmers University of Technology, 41296 Gothenburg, Sweden

Igor O. Sokolov† and Ivano Tavernelli‡

IBM Quantum, IBM Research Zurich, Säumerstrasse 4, 8803 Rüschlikon, Switzerland

Ke Liao§ and Pablo López Ríos

Max Planck Institute for Solid State Research, Heisenbergstr. 1, 70569 Stuttgart, Germany

Ali Alavi¶

Max Planck Institute for Solid State Research, Heisenbergstr. 1, 70569 Stuttgart, Germany and Yusuf Hamied Department of Chemistry, University of Cambridge, Lensfield Road, Cambridge CB2 1EW, United Kingdom

(Dated: May 2, 2024)

Contents

I. Calculation details 2
  A. Beryllium 2
  B. H_2 2
  C. LiH Details 2

II. MP2 Natural Orbitals 3

III. H_2 Complete Basis Set limit 4

IV. LiH Dissociation energy and spectroscopic constants 4

V. LiH QASM simulations / hardware calculations and error mitigation 4
  A. QASM simulations 5
  B. Zero noise extrapolation 6
  C. Reference-state error mitigation 6
  D. Hardware experiments 7

VI. Hardware characteristics 7

VII. The 1-norm of transcorrelated qubit Hamiltonians 8

References 8

* dobrautz@chalmers.se
† Current address: PASQAL, 7 rue Léonard de Vinci, 91300 Massy, France
‡ ita@zurich.ibm.com
§ Current address: Department of Physics, Arnold Sommerfeld Center for Theoretical Physics, Ludwig-Maximilians-Universität München, Theresienstrasse 37, 80333 Munich, Germany
¶ a.alavi@fkf.mpg.de

I. Calculation details

Workflow of the hybrid quantum-classical TC-VarQITE approach to solving for the right eigenvector and groundstate energy of the TC Hamiltonian $\bar{H}$. On a classical computer, we perform a conventional Hartree-Fock and optionally a MP2 calculation, using `PySCF`,[1, 2] or OpenMolcas[3] in a chosen basis set to obtain starting orbitals and $|\phi_0\rangle$. In our applications, we use a Drummond-Towler-Needs Jastrow factor, [4, 5] $\hat{J}$, which we optimize with variational Monte Carlo (VMC)[6–8] (with a scaling of $\mathcal{O}(n_e^3)$ on conventional hardware) using the `CASINO` package.[9, 10] Details on the VMC optimization process can be found in Ref. [8]. We then use the `TCHint` library to calculate the 2- and 3-body integrals required to construct the molecular Hamiltonian in second quantization. Then we enter the hybrid quantum-classical optimization loop, sketched in Fig. 1d of the main text, consisting of preparing a parametrized Ansatz and measuring the gradient and the metric.

Sample input files and scripts for all stages of the workflow and TC Hamiltonians in qubit operator form can be found in the `Github` repository accompanying the paper.[11]

A. Beryllium

For beryllium (Fig. 2 in the main text), we performed all-electron calculations using Hartree-Fock orbitals from different basis sets, shown in Table S1. The conventional no-TC FCI results were obtained with `PySCF`[2] using Dunning's correlation-consistent polarized basis sets[12] of increasing size, with (cc-pCVxZ) and without core functions (cc-pVxZ). Input files for the VMC optimization of the Jastrow factor using `CASINO` can be found in the `Github` repository accompanying the paper.[11] The TC-VarQITE results used much smaller basis sets in the form of a minimal (STO-6G), two Pople basis sets (631G and 6311G) and a larger cc-pVTZ basis set. Table S1 shows the number of spin-orbitals in the corresponding basis set and, assuming a Jordan-Wigner (JW) fermion-to-qubit mapping, the equivalent number of qubits required to represent the problem on quantum hardware. The TC-VarQITE result in an STO-6G basis set (10 qubits) was obtained by noise-free simulation of quantum hardware. In contrast, the larger basis sets (631G, 6311G, and cc-pVTZ) were obtained with a classical solver in the form of the TC-full configuration quantum Monte Carlo (TC-FCIQMC) method.[13–15]

Method	Basis set	Number of spin-orbitals/qubits^a	Energy
no-TC FCI	cc-pVDZ	28	-14.6170
	cc-pVTZ	60	-14.6235
	cc-pVQZ	110	-14.6396
	cc-pCVDZ	36	-14.6515
	cc-pCVTZ	86	-14.6616
	cc-pCVQZ	168	-14.6653
TC-VarQITE	STO-6G	10	-1.46647
	631G	18	-1.46656
	6311G	26	-1.46669
	cc-pVTZ	60	-1.46664

^{a} With Jordan-Wigner encoding

Table S1. Details on the beryllium results shown in Fig. 2 of the main text in the form of the used basis set, the corresponding number of spin-orbitals, and the required number of qubits on quantum hardware (assuming JW encoding). Energies are reported in Hartree.

B. H_2

For H_2 (Fig. 3 in the main text), we performed all-electron calculations using Hartree-Fock orbitals from different basis sets, shown in Table S2, and bond distances. Sample input files for the VMC optimization of the Jastrow factor using `CASINO` can be found in the `Github` repository accompanying the paper.[11] The conventional no-TC FCI results in a cc-pVDZ basis were obtained with `PySCF`.[2] CT-F12 results and CBS limit estimated were obtained from Ref. [16] The TC-VarQITE results in an STO-6G (4 qubits) and 6-31G basis (8 qubits) were obtained by noise-free simulation of quantum hardware, while the cc-pVDZ results (20 qubits) were obtained with TC-FCIQMC.[13–15]

Method	Basis set	Number of spin-orbitals/qubits^a
TC-VarQITE	STO-6G	4
	6-31G	8
	cc-pVDZ	20
no-TC FCI	cc-pVDZ	20

^{a} With Jordan-Wigner encoding

Table S2. Details on the H_2 results shown in Fig. 3 of the main text in the form of the used basis set, the corresponding number of spin-orbitals, and the required number of qubits on quantum hardware (assuming JW encoding). Energies for all bond distances can be found in the `Github` repository accompanying the paper.[11]

C. LiH Details

Except for the results shown in Fig. 4c of the main text, we performed frozen-core calculations with the 1s orbital of Li frozen. For the LiH calculations, we used both HF orbitals (from different basis sets, see Table S3), as well

as MP2 natural orbitals (MP2-NOs) obtained in the cc-pVDZ basis set. The number of used MP2-NOs (from the maximum of 19 spatial orbitals in a cc-pVDZ basis) was truncated based on the natural orbital occupation number. For details on the MP2-NOs, see Section II. We also provide `Python` scripts to obtain the MP2-NOs using `PySCF`[2] in the `Github` repository accompanying the paper.[11] Sample input files for the VMC optimization of the Jastrow factor using `CASINO` can be found in the `Github` repository accompanying the paper.[11] Details on the used methods, type of calculations (all-electron/frozen-core), used orbitals types, basis sets, and the number of orbitals/qubits (depending on the fermion-to-qubit mapping), as well as dissociation energy results of Fig. 4d, can be found in Table S3. CT-F12 results and CBS limit estimated were obtained from Ref. [16] Conventional no-TC FCI results were obtained with `PySCF`.[2] Noise-free TC-VarQITE simulations up to 12 qubits were performed using a matrix-based statevector simulator based on `Qiskit`.[17] In contrast, larger calculations were performed with the TC-FCIQMC method. For details on the noisy simulations (QASM) and actual hardware experiments (HW), as well as error mitigation schemes, see Section V and the main text Methods.

Table S3. LiH details

Figure	Method	Orbital-type	# **spatial** orbitals	Encoding	# qubits	Energy[c]
4a	Frozen-core no-TC FCI	HF	19 (cc-pVDZ)	JW	38	[a]
	Frozen-core TC-VarQITE	MP2-NO	3	JW	6	[a]
	Frozen-core TC-VarQITE	MP2-NO	4	JW	8	[a]
4c	All-electron FCI/TC-VarQITE	MP2-NO	14	JW	28	–
4d	Frozen-core no-TC FCI	HF	19 (cc-pVDZ)	JW	38	81.66
	Frozen-core no-TC FCI	HF	43 (cc-pVTZ)	JW	86	89.59
	Frozen-core TC-VarQITE	HF	5 (STO-6G)	JW	10	59.39
	Frozen-core TC-VarQITE	HF	10 (631G)	JW	20	85.01
	Frozen-core TC-VarQITE	HF	19 (cc-pVDZ)	JW	38	88.88
	Frozen-core TC-VarQITE	MP2-NO	3	JW	6	88.54
	Frozen-core TC-VarQITE	MP2-NO	4	JW	8	90.35
	Frozen-core TC-VarQITE	MP2-NO	5	JW	10	89.51
	Frozen-core TC-VarQITE	MP2-NO	6	JW	12	89.56
	Frozen-core TC-VarQITE QASM[b]	MP2-NO	3	Parity	4	-22(20)
	Frozen-core TC-VarQITE HW[b]	MP2-NO	3	Parity	4	-35(7)
	Frozen-core TC-VarQITE QASM/ZNE[b]	MP2-NO	3	Parity	4	95(62)
	Frozen-core TC-VarQITE QASM/REM[b]	MP2-NO	3	Parity	4	87(40)
	Frozen-core TC-VarQITE HW/REM[b]	MP2-NO	3	Parity	4	89(31)

[a] See `Github` repository.[11][b] QASM/Hardware results with parity encoding and subsequent two-qubit reduction. HW with REM results are shown here, for other results see below. [c] Total energies for Fig. 4a in Hartree, dissociation energies for Fig. 4d in mH.

II. MP2 Natural Orbitals

Box I sketches the motivation behind using MP2-NOs:

Box I

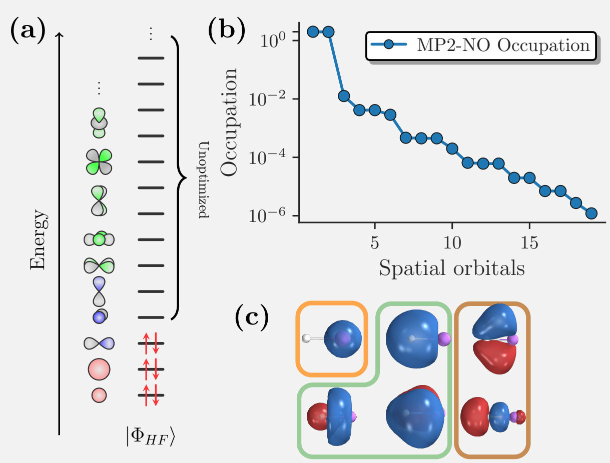

The motivation behind using more optimized orbitals, like MP2 natural orbitals. (a) The VMC optimization of the Jastrow factor, $E_{\mathrm{VMC}} = \langle \Phi_{\mathrm{HF}} | \hat{H} | \Phi_{\mathrm{HF}} \rangle$, only takes into account the occupied orbitals in the initial HF solution, while the vast number of virtual orbitals, from, e.g., commonly used basis sets as the correlation-consistent family,[12] are not optimized for the TC method. (b) Occupation of the cc-pVDZ basis set-derived MP2 NOs of LiH at the equilibrium bond distance. The NO occupation allows an intuitive and well-motivated truncation of the virtual orbital space. (c) All relevant orbitals at the LiH equilibrium bond distance. The orange box shows the lowest energy orbital, which exclusively is of Li 1s character and frozen in some subsequent calculations. The green box depicts the next three most important/occupied MP2 NOs defined as the active space in most of our calculations. The brown box encompasses two additional orbitals used for the 12(10) in the all-electron(frozen-core) calculations respectively.

Figure S1a shows the TC all-electron total energies of LiH at equilibrium geometry as a function of the number of used orbitals (HF and MP2 natural orbitals) in a cc-pVDZ basis. When truncating the number of utilized virtual orbitals, the total energy monotonically increases when using HF orbitals. With MP2-NOs, there is a slight non-

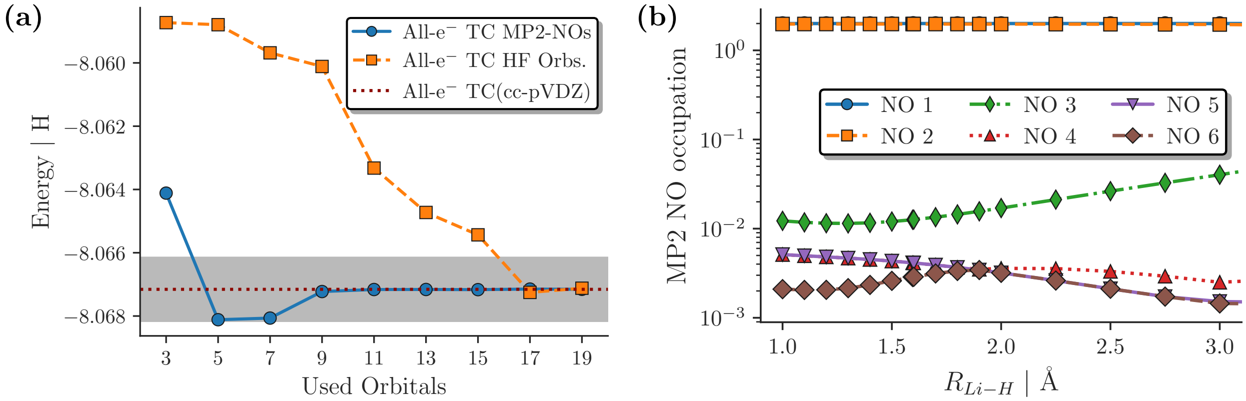

FIG. S1. (a) Convergence of all-electron total energies of LiH at equilibrium using MP2 NOs in a cc-pVDZ basis as a function of the number of utilized orbitals (HF/MP2-NOs). (b) VDZ MP2 NO Occupation vs. R

monotonic behavior in the total energy when using fewer orbitals. However, the non-monotonic behavior is small, only within 1 kcal/mol (gray area) of our reference cc-pVDZ results (red dotted line, using all orbitals). Overall the total energy computed using MP2-NOs is close to the reference result using the full number of orbitals of the basis set (red dotted line). Figure S1a shows the LiH MP2-NO occupation of the six most occupied orbitals as a function of bond distance within a cc-pVDZ basis. For frozen-core calculations, we included the 3 and 4 most occupied MP2-NOs.

III. H_2 Complete Basis Set limit

Figure S2 shows conventional FCI (no-TC) and TC-FCI calculations for H_2 at 0.7 Å as a function of basis set size/number of spin-orbitals/qubits. The basis sets used are 6-31G and cc-pVDZ for TC calculations and additionally cc-pVTZ and cc-pVQZ for no-TC calculations. The CBS estimate is taken from Ref. [16].

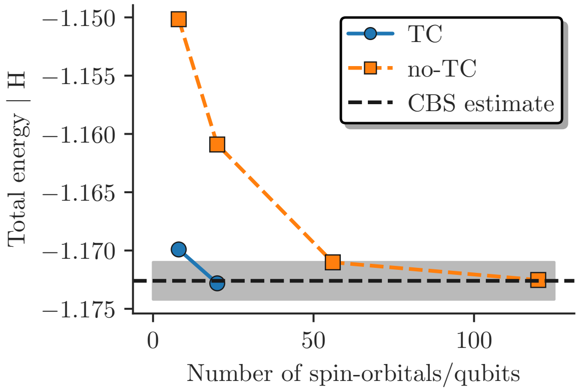

FIG. S2. H_2 TC results compared with conventional FCI results vs. basis set.

IV. LiH Dissociation energy and spectroscopic constants

We obtained the LiH dissociation energy estimates as the difference of the frozen-core total energy of the LiH molecule at equilibrium geometry of 1.595 Å[18] and the sum of the energies of isolated hydrogen and lithium atoms. The CBS-limit total energy of hydrogen was taken as -0.5 H.[19] For lithium, we calculated the frozen-core TC CBS limit by extrapolation of the HF energy (frozen-core Li is a one-electron problem) up to the cc-pV5Z basis set. To do so, we fitted cc-pVxZ results to an exponential, $a + b\,\mathrm{e}^{-cx}$, as is common practice for HF energies.[20] The frozen-core Li CBS energy resulting from this procedure is -7.432713 H.

We calculated the remaining spectroscopic constants (equilibrium distances and vibrational frequencies) of H_2 and LiH by calculating multiple total energy values around the respective equilibrium bond distance, see Fig. S4, and fitting a quadratic function, $f(R) = a(R-b)^2 + c$, to the results, where R is the bond distance. We obtained the equilibrium distance, R_e from the fit-parameter b and the vibrational frequency, ω_e, from a. The calculations and fit details can be found in Fig. S4.

V. LiH QASM simulations / hardware calculations and error mitigation

Our quantum assembly language (QASM)[21] simulations of the `ibm_lagos` device include statistical measurement noise and a dedicated hardware noise model. Both hardware experiments and QASM simulations employ readout error mitigation[22] as implemented in Qiskit and use a hardware-efficient Ansatz. For details about the device and

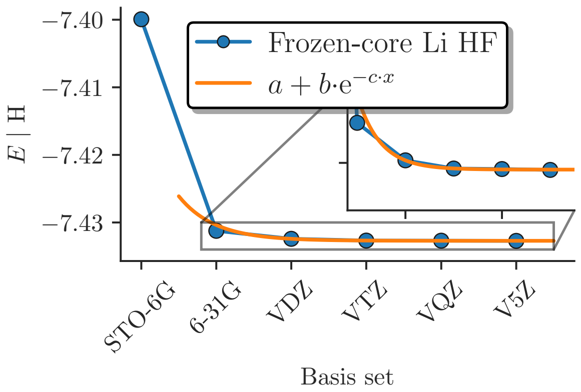

FIG. S3. Frozen-core Li HF energies for increasing basis set size. The orange line shows the exponential fit to the cc-pVxZ basis set results.

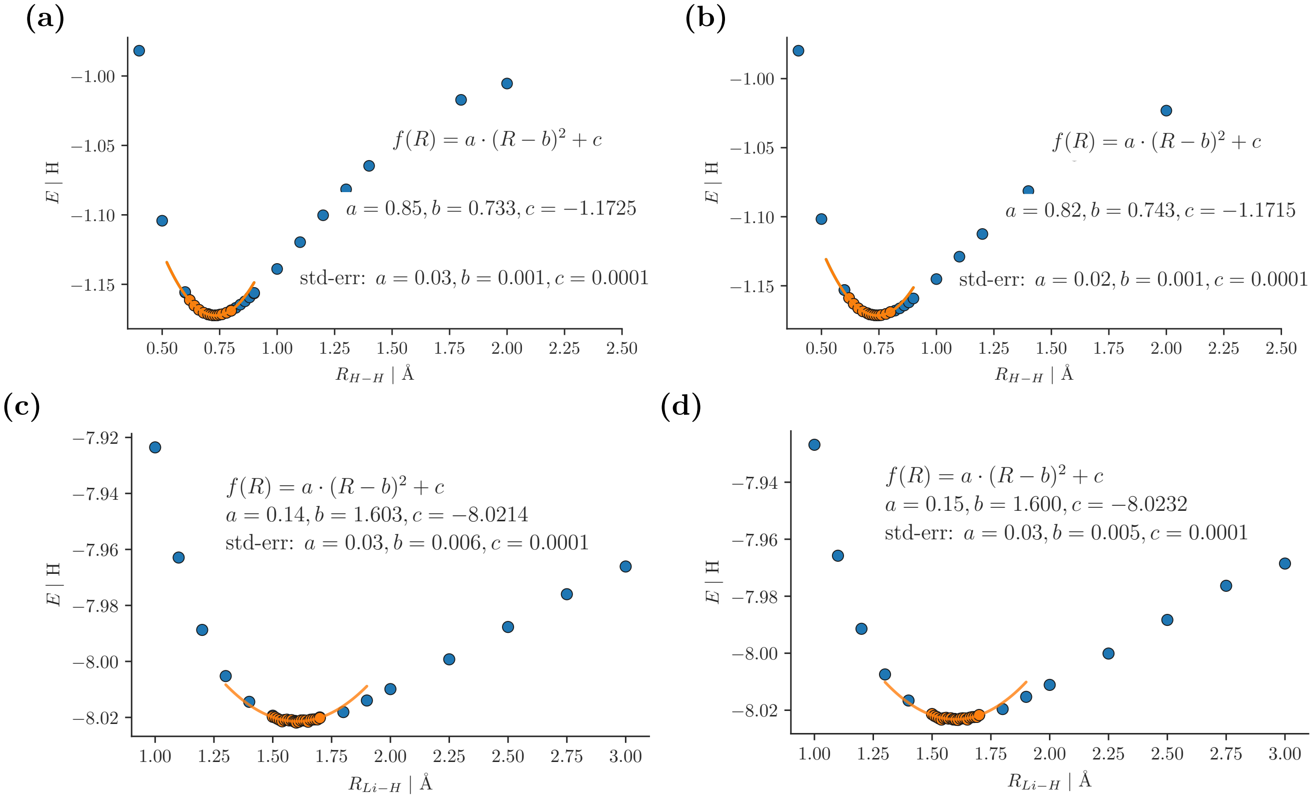

FIG. S4. TC total energies as a function of bond distance for (a) H_2 (STO-6G/HF orbitals, two qubits with parity encoding and two-qubit reduction) (b) H_2 (631-G/HF orbitals, 6 qubits with parity encoding and two-qubit reduction), (c) LiH (cc-pVDZ/3 MP2-NO, four qubits with parity encoding and two-qubit reduction) and (d) LiH (cc-pVDZ/4 MP2-NO, 6 qubits with parity encoding and two-qubit reduction). The orange data points indicate the values considered in the quadratic fit, which is shown by the orange line. Results of the fit and standard errors of the fitted parameters are indicated in the figures.

its noise model, see below. All quantum simulations and experiments are performed with Qiski.t[17]

In this section, we disclose details of hardware experiments and QASM simulations used to evaluate the dissociation energy of LiH. Two kinds of error mitigation schemes were evaluated separately: zero noise extrapolation (ZNE)[23, 24] and reference error mitigation (REM).[25] We calculated the frozen-core energy of LiH at equilibrium geometry (1.595 Å) using 3 MP2-NOs with QASM simulations using the noise model of and with actual experiments on the IBMQ `ibm_lagos` device, see Tables S7 and S8. We employed readout error mitigation,[22] parity encoding, and a (two layers if not stated otherwise) R_y ansatz with a linear `CNOT` entangling layer, see Fig. 5c of the main text.

A. QASM simulations

Ten independent QASM VarQITE simulations with the noise model of the `ibm_lagos` device were run, each using 50000 shots for every measurement of the gradient, Fischer information matrix, and energy terms. We initialized the R_y gate parameters to start from the Hartree-Fock state and averaged the energy expectation values after VarQITE

convergence (after 60 iterations for all ten independent runs). The initial energy (HF energy) and the average converged energies are listed in Table S4. Table S4 also lists the mean HF energy.

Table S4. Initial (HF) LiH QASM energies and averaged converged energies after 60 iterations. Shot-based standard deviations are indicated by the uncertainty in the last digit in parentheses for HF calculations. The standard deviations of all energy measurements are about 0.002 H, so the parentheses for the averaged converged energies indicate the standard deviations of the mean value after 60 VarQITE iterations. We also show the exact HF energy and the resulting REM correction, $\Delta E_{\mathrm{REM}} = E_{\mathrm{HF}}^{\mathrm{exact}} - E_{\mathrm{HF}}^{\mathrm{VarQITE}}$, as well as the REM-corrected final mean energy values, $\bar{E} + \Delta E_{\mathrm{RM}}$.

Run	HF energy	$\bar{E}$
0	-7.879(2)	-7.883(2)
1	-7.891(2)	-7.899(2)
2	-7.888(2)	-7.892(3)
3	-7.904(2)	-7.902(2)
4	-7.908(2)	-7.916(2)
5	-7.880(2)	-7.883(2)
6	-7.895(2)	-7.894(2)
7	-7.932(2)	-7.942(2)
8	-7.930(2)	-7.928(1)
9	-7.934(2)	-7.941(2)
Mean	-7.90(2)	-7.91(2)
Exact	-8.0186912148	-8.021612
REM correction	-0.12(2)	—
REM-corrected	—	-8.02(4)

B. Zero noise extrapolation

In zero noise extrapolation (ZNE), the noise of a QPU is artificially increased by a factor α either by a repetition of gates on actual hardware or numerically in QASM simulations. The measurements of interest at different noise amplifications, i.e., $E(\alpha)$, are then extrapolated to a zero-noise estimate $E(\alpha = 0)$.

Zero noise extrapolation was performed on top of our QASM simulations by artificially increasing the noise parameters (readout error, probability to measure 0/1 when prepared in 1/0) of the simulated qubits by a numerical factor α, and decreased the T_1 and T_2 times by a factor of $1/\alpha$. We chose the commonly used factors of $\alpha = 3, 5, 7$ and 9, since these values of noise amplification can also be mimicked on hardware by increasing the number of 2-qubit gates (dominant error sources) by these multiplicative numbers. Fig. S5 shows the error with respect to the exact frozen-core LiH energy at $R = 1.595$ Å in a cc-pVDZ basis set. We fitted a linear function to the first three data points of the original and noise-amplified results. The ZNE error is $-0.007(4)$ H.

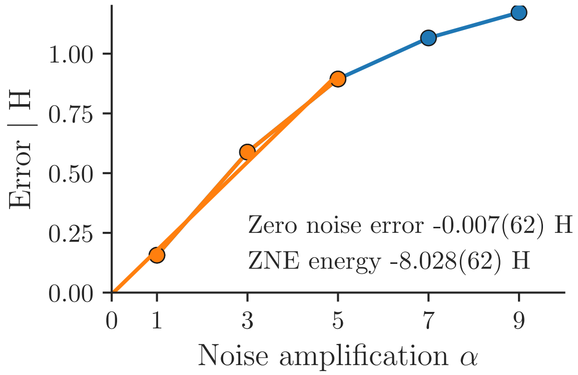

FIG. S5. Zero noise extrapolation of frozen-core LiH QASM results at equilibrium geometry, $R = 1.595$ Å.

C. Reference-state error mitigation

More details on the performed ZNE for the LiH results can be found below.

Reference-state error mitigation (REM) is a recently developed quantum error mitigation scheme,[25] which accurately estimates the total energy error for a proxy problem, a reference wave function. The reference state is chosen such that its energy can be efficiently calculated (exactly) on classical hardware. After preparation of the reference state $|\Phi(\boldsymbol{\theta}_{\mathrm{ref}})\rangle$ on quantum hardware, one can determine the resulting energy error ΔE_{REM} at the reference parameters, $\boldsymbol{\theta}_{\mathrm{ref}}$,

$$\Delta E_{\mathrm{REM}} = E_{\mathrm{QPU}}(\boldsymbol{\theta}_{\mathrm{ref}}) - E_{\mathrm{CPU}}(\boldsymbol{\theta}_{\mathrm{ref}}), \tag{1}$$

where $E_{\mathrm{CPU}}(\boldsymbol{\theta}_{\mathrm{ref}})$ is the classically evaluated (noiseless) energy of the reference state. $E_{\mathrm{QPU}}(\boldsymbol{\theta}_{\mathrm{ref}})$ is the noisy energy measured on the quantum device at the reference parameter values, $\boldsymbol{\theta}_{\mathrm{ref}}$. The noiseless (exact) energy at arbitrary parameters, $E_{\mathrm{exact}}(\boldsymbol{\theta})$, can be expressed as

$$E_{\mathrm{exact}}(\boldsymbol{\theta}) = E_{\mathrm{QPU}}(\boldsymbol{\theta}) - \Delta E_{\mathrm{REM}} - \Delta E_p(\boldsymbol{\theta}), \tag{2}$$

where $\Delta E_p(\boldsymbol{\theta})$ describes any parameter-dependence of noise, which vanishes at the reference parameters, $\Delta E_p(\boldsymbol{\theta}_{\text{ref}}) = 0$. In other words, the validity of REM depends on the Euclidean distance of the reference state to the exact solution $|\boldsymbol{\theta}_{\text{exact}} - \boldsymbol{\theta}_{\text{ref}}|$, in the parameter space $\{\boldsymbol{\theta}\}$, and the degree to which the noise is significantly different for the two wavefunctions. In this work, ΔE_{REM} is used to correct the final TC-VarQITE energy measurements following Eq. (2). The reference parameter values $\boldsymbol{\theta}_{\text{ref}}$ here refer to the Hartree-Fock (HF, mean-field) wavefunction. The HF state is a particularly suitable error-mitigation reference for molecular systems that do not have strong multireference character, which is the case for the HW experiments described herein. The suitability of this choice of a reference state is increased using the TC approach since the HF coefficient of the right-eigenvector solution is enhanced compared to the non-TC case (see Fig. 4c of the main text). Because we are using the same HF reference state also as the initial state for TC-VarQITE, REM does not impose any computational overhead. The energy measurements of the initial TC-VarQITE iteration step suffice to determine the REM correction, ΔE_{REM}.

D. Hardware experiments

Hardware experiments were run on `ibm_lagos`. These calculations enforced a frozen-core approximation for LiH and concerned the equilibrium bond length of $R_e = 1.595$ Å. Due to the severe influence of hardware noise and long time-to-solution, we used only a single layer R_y ansatz, as shown in Fig. 5c of the main text, and 10000 shots. The dissociation energy of LiH was estimated by first measuring the HF energy on `ibm_lagos` and subsequently calculating the REM correction (see methods section of main text). We then measured and REM-corrected the final VarQITE energy using gate parameters obtained from an exact statevector simulation. The exact gate parameters are given in Table S5. Each hardware experiment was repeated five times, both to calculate the HF and final VarQITE energy, respectively. The results of these measurements are summarized in Table. S6.

Table S5. Final VarQITE gate parameters for a 1-layer, linear entangled R_y ansatz as shown in Fig. 5c of the main text.

Gate #	0	1	2	3	4	5	6	7
Gate angle	-3.347357	-3.145617	0.0	0.0	3.180561	3.141593	-0.003530	0.0

VI. Hardware characteristics

Table S7 presents the device characteristics used for the noise model in our QASM simulations, which were obtained during our hardware experiments. This information includes T1 and T2 relaxation times, qubit frequencies, readout errors, and error rates for single-qubit and two-qubit gates per qubit. These parameters are necessary to recreate our noise model using Qiskit. To construct the noise model of the `ibm_lagos`, we followed the same procedure as in Ref.[26], which is summarized below. Our QASM simulations considered depolarization, thermalization, and readout errors. The depolarization error was modeled as the decay of the noiseless density matrix $\rho = |\Phi\rangle\langle\Phi|$ to the uncorrelated density matrix $\mathbf{1}/2^{N_q}$ as

$$\rho_d = \gamma_1 \text{Tr}[\rho]\mathbf{1}/2^{N_q} + (1-\gamma_1)\,\rho, \tag{3}$$

with N_q being the number of qubits and γ_1 representing the decay rate, which was estimated using gate fidelities provided in Tab. S7. The thermalization error of a qubit, which consists of general amplitude dampening and phase flip error, was defined as the decay towards the Fermi-Dirac distribution of ground and excited states based on their energy difference ω:

$$\rho_t = p|0\rangle\langle 0| + (1-p)|1\rangle\langle 1|, \tag{4}$$

with $p = (e^{\frac{-\omega}{k_b T}} + 1)^{-1}$, T being the temperature and k_B, the Boltzmann constant. Finally, the readout error was modeled by calibrating the measurement error matrix $\mathcal{M}$, which assigns a probability to readout all possible states for any N_q-qubit computational basis state. In an ideal, noiseless situation, $\mathcal{M}$ would be characterized by $\mathcal{P}(i|j) = 1$ for $i = j$ and $\mathcal{P}(i|j) = 0$ for $i \neq j$.

Table S6. Initial (HF) LiH hardware energy results and results obtained using the exact VarQITE GS parameters for a 1-layer R_y ansatz and linear entanglement given in Table S5.

Run	HF energy	Final energy
0	-7.8838	-7.9020
1	-7.9031	-7.8713
2	-7.9012	-7.9238
3	-7.9001	-7.8768
4	-7.8979	-7.9289
Mean	-7.897(7)	-7.901(24)
REM correction	-0.122(7)	—
REM-corrected	—	-8.022(31)

Table S7. Characteristics of the `ibm_lagos` at the time of hardware and noisy QASM simulations. Data for the calibration date of 22/2/2023.

Qubit	T1 (us)	T2 (us)	Frequency (GHz)	Anharmonicity (GHz)	Readout assignment error	Prob meas0 prep1	Prob meas1 prep0	Readout length (ns)	ID error	$\sqrt{x}$ (sx) error	Pauli-X error
0	190.49	43.28	5.24	-0.340	0.021	0.020	0.022	789.33	0.00032	0.00032	0.00032
1	101.53	98.26	5.10	-0.343	0.021	0.025	0.018	789.33	0.00023	0.00023	0.00023
2	121.44	124.84	5.19	-0.342	0.011	0.013	0.008	789.33	0.00016	0.00016	0.00016
3	165.16	88.69	4.99	-0.345	0.013	0.016	0.011	789.33	0.00018	0.00018	0.00018
4	99.89	30.01	5.29	-0.339	0.026	0.025	0.027	789.33	0.00021	0.00021	0.00021
5	139.52	77.89	5.18	-0.341	0.015	0.016	0.015	789.33	0.00029	0.00029	0.00029
6	165.11	72.85	5.06	-0.343	0.011	0.013	0.009	789.33	0.00039	0.00039	0.00039

Table S8. Characteristics of the `ibm_lagos` at the time of hardware and noisy QASM simulations. Data for the calibration date of 22/2/2023. The notation "0_1" denotes the CNOT gate between qubits 0 (control) and 1 (target).

Qubit	CNOT error	Gate time (ns)
0	**0_1:** 0.0112	**0_1:** 576
1	**1_3:** 0.0056; **1_2:** 0.0075; **1_0:** 0.0112	**1_3:** 334; **1_2:** 327; **1_0:** 611
2	**2_1:** 0.0075	**2_1:** 291
3	**3_1:** 0.0056; **3_5:** 0.0073	**3_1:** 299; **3_5:** 334
4	**4_5:** 0.0074	**4_5:** 363
5	**5_4:** 0.0074; **5_6:** 0.0080; **5_3:** 0.0073	**5_4:** 327; **5_6:** 256; **5_3:** 299
6	**6_5:** 0.0080	**6_5:** 292

VII. The 1-norm of transcorrelated qubit Hamiltonians

Table S9 contains the normalized 1-norms (in percent) of the the coefficients, c_i, of the linear combination of unitaries decompositions for molecular electronic structure Hamiltonians,

$$\hat{H} = \sum c_i \hat{P}_i, \tag{5}$$

corresponding to 0-, 1-, 2- and 3-body operators of LiH in a STO-6G basis set (12 qubits) at 3 bond distances.

Table S9. table

Normalized 1-norms (sum of absolute values, $\sum_i |c_i|$) of the coefficients of the qubit operator (in percent) corresponding to 0-, 1-, 2- and 3-body operators in Eq. 6 of LiH in a STO-6G basis set (12 qubits) at 3 bond distances (in Å), with (TC) and without transcorrelation (no-TC). We note, that the no-TC Hamiltonian has no 3-body operators.

	no-TC			TC			
Bond distance	0-body	1-body	2-body	0-body	1-body	2-body	**3-body**
1.60	85.34	9.14	5.52	79.92	12.57	7.48	**0.03**
2.25	83.40	10.57	6.03	78.82	13.24	7.90	**0.03**
3.00	80.81	12.20	6.99	74.42	16.76	8.75	**0.07**

Bibliography

[1] Q. Sun, T. C. Berkelbach, N. S. Blunt, G. H. Booth, S. Guo, Z. Li, J. Liu, J. D. McClain, E. R. Sayfutyarova, S. Sharma, S. Wouters, and G. K. Chan, Pyscf: the python-based simulations of chemistry framework, WIREs Computational Molecular Science **8**, 10.1002/wcms.1340 (2017).

[2] Q. Sun, X. Zhang, S. Banerjee, P. Bao, M. Barbry, N. S. Blunt, N. A. Bogdanov, G. H. Booth, J. Chen, Z.-H. Cui, J. J. Eriksen, Y. Gao, S. Guo, J. Hermann, M. R. Hermes, K. Koh, P. Koval, S. Lehtola, Z. Li, J. Liu, N. Mardirossian, J. D. McClain, M. Motta, B. Mussard, H. Q. Pham, A. Pulkin, W. Purwanto, P. J. Robinson, E. Ronca, E. R. Sayfutyarova, M. Scheurer, H. F. Schurkus, J. E. T. Smith, C. Sun, S.-N. Sun, S. Upadhyay, L. K. Wagner, X. Wang, A. White, J. D. Whitfield, M. J. Williamson, S. Wouters, J. Yang, J. M. Yu, T. Zhu, T. C. Berkelbach, S. Sharma, A. Y. Sokolov, and G. K.-L. Chan, Recent developments in the pySCF program package, The Journal of Chemical Physics **153**, 024109 (2020).

[3] G. Li Manni, I. Fdez. Galván, A. Alavi, F. Aleotti, F. Aquilante, J. Autschbach, D. Avagliano, A. Baiardi, J. J. Bao, S. Battaglia, L. Birnoschi, A. Blanco-González, S. I. Bokarev, R. Broer, R. Cacciari, P. B. Calio, R. K. Carlson, R. Carvalho Couto, L. Cerdán, L. F. Chibotaru, N. F. Chilton, J. R. Church, I. Conti, S. Coriani, J. Cuéllar-Zuquin, R. E.

Daoud, N. Dattani, P. Decleva, C. de Graaf, M. G. Delcey, L. De Vico, W. Dobrautz, S. S. Dong, R. Feng, N. Ferré, M. Filatov(Gulak), L. Gagliardi, M. Garavelli, L. González, Y. Guan, M. Guo, M. R. Hennefarth, M. R. Hermes, C. E. Hoyer, M. Huix-Rotllant, V. K. Jaiswal, A. Kaiser, D. S. Kaliakin, M. Khamesian, D. S. King, V. Kochetov, M. Krośnicki, A. A. Kumaar, E. D. Larsson, S. Lehtola, M.-B. Lepetit, H. Lischka, P. López Ríos, M. Lundberg, D. Ma, S. Mai, P. Marquetand, I. C. D. Merritt, F. Montorsi, M. Mörchen, A. Nenov, V. H. A. Nguyen, Y. Nishimoto, M. S. Oakley, M. Olivucci, M. Oppel, D. Padula, R. Pandharkar, Q. M. Phung, F. Plasser, G. Raggi, E. Rebolini, M. Reiher, I. Rivalta, D. Roca-Sanjuán, T. Romig, A. A. Safari, A. Sánchez-Mansilla, A. M. Sand, I. Schapiro, T. R. Scott, J. Segarra-Martí, F. Segatta, D.-C. Sergentu, P. Sharma, R. Shepard, Y. Shu, J. K. Staab, T. P. Straatsma, L. K. Sørensen, B. N. C. Tenorio, D. G. Truhlar, L. Ungur, M. Vacher, V. Veryazov, T. A. Voß, O. Weser, D. Wu, X. Yang, D. Yarkony, C. Zhou, J. P. Zobel, and R. Lindh, The openmolcas web: A community-driven approach to advancing computational chemistry, Journal of Chemical Theory and Computation **19**, 6933–6991 (2023).

[4] N. D. Drummond, M. D. Towler, and R. J. Needs, Jastrow correlation factor for atoms, molecules, and solids, Phys. Rev. B **70**, 235119 (2004).

[5] P. López Ríos, P. Seth, N. D. Drummond, and R. J. Needs, Framework for constructing generic Jastrow correlation factors, Phys. Rev. E **86**, 036703 (2012).

[6] D. Ceperley, G. V. Chester, and M. H. Kalos, Monte Carlo simulation of a many-Fermion study, Phys. Rev. B **16**, 3081 (1977).

[7] W. M. C. Foulkes, L. Mitas, R. J. Needs, and G. Rajagopal, Quantum monte carlo simulations of solids, Rev. Mod. Phys. **73**, 33 (2001).

[8] J. P. Haupt, S. M. Hosseini, P. L. Ríos, W. Dobrautz, A. Cohen, and A. Alavi, Optimizing jastrow factors for the transcorrelated method, The Journal of Chemical Physics **158**, 10.1063/5.0147877 (2023).

[9] R. J. Needs, M. D. Towler, N. D. Drummond, P. L. Ríos, and J. R. Trail, Variational and diffusion quantum Monte Carlo calculations with the CASINO code, J. Chem. Phys. **152**, 154106 (2020).

[10] P. López Ríos, A. Ma, N. D. Drummond, M. D. Towler, and R. J. Needs, Inhomogeneous backflow transformations in quantum monte carlo calculations, Phys. Rev. E **74**, 066701 (2006).

[11] W. Dobrautz, tc-varqite-hamiltonians (2023).

[12] T. H. Dunning, Gaussian basis sets for use in correlated molecular calculations. I. The atoms boron through neon and hydrogen, J. Chem. Phys. **90**, 1007 (1989).

[13] W. Dobrautz, H. Luo, and A. Alavi, Compact numerical solutions to the two-dimensional repulsive hubbard model obtained via nonunitary similarity transformations, Phys. Rev. B **99**, 075119 (2019).

[14] K. Guther, R. J. Anderson, N. S. Blunt, N. A. Bogdanov, D. Cleland, N. Dattani, W. Dobrautz, K. Ghanem, P. Jeszenszki, N. Liebermann, G. L. Manni, A. Y. Lozovoi, H. Luo, D. Ma, F. Merz, C. Overy, M. Rampp, P. K. Samanta, L. R. Schwarz, J. J. Shepherd, S. D. Smart, E. Vitale, O. Weser, G. H. Booth, and A. Alavi, NECI: N-electron configuration interaction with an emphasis on state-of-the-art stochastic methods, J. Chem. Phys. **153**, 034107 (2020).

[15] A. J. Cohen, H. Luo, K. Guther, W. Dobrautz, D. P. Tew, and A. Alavi, Similarity transformation of the electronic Schrödinger equation via Jastrow factorization, J. Chem. Phys. **151**, 061101 (2019).

[16] M. Motta, T. P. Gujarati, J. E. Rice, A. Kumar, C. Masteran, J. A. Latone, E. Lee, E. F. Valeev, and T. Y. Takeshita, Quantum simulation of electronic structure with a transcorrelated Hamiltonian: improved accuracy with a smaller footprint on the quantum computer, Phys. Chem. Chem. Phys. **22**, 24270 (2020).

[17] G. Aleksandrowicz, T. Alexander, P. Barkoutsos, L. Bello, Y. Ben-Haim, D. Bucher, F. J. Cabrera-Hernández, J. Carballo-Franquis, A. Chen, C.-F. Chen, J. M. Chow, A. D. Córcoles-Gonzales, A. J. Cross, A. Cross, J. Cruz-Benito, C. Culver, S. D. L. P. González, E. D. L. Torre, D. Ding, E. Dumitrescu, I. Duran, P. Eendebak, M. Everitt, I. F. Sertage, A. Frisch, A. Fuhrer, J. Gambetta, B. G. Gago, J. Gomez-Mosquera, D. Greenberg, I. Hamamura, V. Havlicek, J. Hellmers, Łukasz Herok, H. Horii, S. Hu, T. Imamichi, T. Itoko, A. Javadi-Abhari, N. Kanazawa, A. Karazeev, K. Krsulich, P. Liu, Y. Luh, Y. Maeng, M. Marques, F. J. Martín-Fernández, D. T. McClure, D. McKay, S. Meesala, A. Mezzacapo, N. Moll, D. M. Rodríguez, G. Nannicini, P. Nation, P. Ollitrault, L. J. O'Riordan, H. Paik, J. Pérez, A. Phan, M. Pistoia, V. Prutyanov, M. Reuter, J. Rice, A. R. Davila, R. H. P. Rudy, M. Ryu, N. Sathaye, C. Schnabel, E. Schoute, K. Setia, Y. Shi, A. Silva, Y. Siraichi, S. Sivarajah, J. A. Smolin, M. Soeken, H. Takahashi, I. Tavernelli, C. Taylor, P. Taylour, K. Trabing, M. Treinish, W. Turner, D. Vogt-Lee, C. Vuillot, J. A. Wildstrom, J. Wilson, E. Winston, C. Wood, S. Wood, S. Wörner, I. Y. Akhalwaya, and C. Zoufal, Qiskit: An Open-source Framework for Quantum Computing (2019).

[18] F. Lovas, Diatomic spectral database, nist standard reference database 114 (2002).

[19] E. R. Davidson, S. A. Hagstrom, S. J. Chakravorty, V. M. Umar, and C. F. Fischer, Ground-state correlation energies for two- to ten-electron atomic ions, Phys. Rev. A **44**, 7071 (1991).

[20] T. Helgaker, P. Jørgensen, and J. Olsen, *Molecular Electronic-Structure Theory* (Wiley, 2000).

[21] A. W. Cross, L. S. Bishop, J. A. Smolin, and J. M. Gambetta, Open quantum assembly language, (2017), arXiv:1707.03429 [quant-ph].

[22] S. Bravyi, S. Sheldon, A. Kandala, D. C. Mckay, and J. M. Gambetta, Mitigating measurement errors in multiqubit experiments, Phys. Rev. A **103**, 042605 (2021).

[23] Y. Li and S. C. Benjamin, Efficient variational quantum simulator incorporating active error minimization, Physical Review X **7**, 10.1103/physrevx.7.021050 (2017).

[24] T. Giurgica-Tiron, Y. Hindy, R. LaRose, A. Mari, and W. J. Zeng, Digital zero noise extrapolation for quantum error mitigation, in *2020 IEEE International Conference on Quantum Computing and Engineering (QCE)* (IEEE, 2020).

[25] P. Lolur, M. Skogh, W. Dobrautz, C. Warren, J. Biznárová, A. Osman, G. Tancredi, G. Wendin, J. Bylander, and M. Rahm, Reference-state error mitigation: A strategy for high accuracy quantum computation of chemistry, Journal of Chemical Theory and Computation **19**, 783 (2023).

[26] I. O. Sokolov, P. K. Barkoutsos, L. Moeller, P. Suchsland, G. Mazzola, and I. Tavernelli, Microcanonical and finite-temperature ab initio molecular dynamics simulations on quantum computers, Phys. Rev. Res. **3**, 013125 (2021).
